# First detection and genetic characterization of canine bufavirus in domestic dogs, Thailand

**DOI:** 10.1038/s41598-024-54914-z

**Published:** 2024-02-27

**Authors:** Kamonpan Charoenkul, Yu Nandi Thaw, Eaint Min Phyu, Waleemas Jairak, Chanakarn Nasamran, Ekkapat Chamsai, Supassama Chaiyawong, Alongkorn Amonsin

**Affiliations:** 1 Faculty of Veterinary Science, Center of Excellence for Emerging and Re-Emerging Infectious Diseases in Animals, Chulalongkorn University, Bangkok, Thailand; 2 Department of Veterinary Public Health, Faculty of Veterinary Science, Chulalongkorn University, Bangkok, 10330 Thailand

**Keywords:** Canine bufavirus, Characterization, Dog, Genetic, Thailand, Molecular biology, Zoology, Diseases

## Abstract

Canine bufavirus (CBuV) was reported in domestic dogs worldwide. We conducted a survey of canine bufavirus in domestic dogs in Thailand from September 2016 to October 2022. Rectal swab samples (n = 531) were collected from asymptomatic dogs and dogs with gastroenteritis signs. The samples were tested for CBuV using PCR with specific primers to the VP1/VP2 gene, and 9.42% (50/531) was CBuV positive. Our findings showed that CBuVs could be detected in both symptomatic and healthy dogs. The Thai CBuVs were found in dogs from different age groups, with a significant presence in those under 1 year (12.60%) and dogs aged 1–5 years (7.34%) (*p* < 0.05), suggesting a high prevalence of Thai CBuVs in dogs under 5 years of age. We performed complete genome sequencing (n = 15) and partial VP1/VP2 sequencing (n = 5) of Thai CBuVs. Genetic and phylogenetic analyses showed that whole genomes of Thai CBuVs were closely related to Chinese and Italian CBuVs, suggesting the possible origin of Thai CBuVs. The analysis of VP1 and VP2 genes in Thai CBuVs showed that 18 of them were placed in subgroup A, while only 2 belonged to subgroup B. This study is the first to report the detection and genetic characterization of CBuVs in domestic dogs in Thailand. Additionally, surveillance and genetic characterization of CBuVs in domestic animals should be further investigated on a larger scale to elucidate the dynamic, evolution, and distribution of CBuVs.

## Introduction

Bufavirus (BuV) is a small, non-enveloped, non-segmented, single-stranded linear DNA virus with a genome size of 4.5–4.8 kb. The BuV is a novel virus of the family *Parvoviridae,* genus Protoparvovirus, which are common pathogens in many animals, including birds and mammals^1^. It contains 2 major open reading frames: ORF1 encoding nonstructural protein 1 (NS1) and ORF2 encoding viral structural protein 1 and 2 (VP1 and VP2)^2^. BuV was first reported in the fecal samples from children with diarrhea in Burkina Faso in 2012^3^. Subsequently, BuV infections have been reported in diarrhea patients in several countries such as Bhutan, China, France, Finland, Netherlands, South Africa, Thailand and Turkey^4–11^. The occurrence of bufavirus infection in humans in those counties ranges from 0.3 to 4%^5,12,13^. These studies speculated that BuV might cause gastroenteritis in human^6,7,14^ BuV is not strictly found in humans but it has also been reported in several animal species such as rats, shrews, pigs, dogs, bats, and primates^15–19^**.**

In 2016, the first report of Canine Bufavirus (CBuV) in dogs in Italy revealed its presence in both dogs with gastroenteric and respiratory symptoms, as well as in asymptomatic dogs. The study reported that the genome of CBuV was closely related to human bufavirus (HBuV)^20^. However, CBuV was genetically distinct from other canine enteric viruses of the *Parvoviridae* family (canine bocavirus (CBoV) and canine parvovirus type 1 and 2 (CPV-1and CPV-2))^21,22^. After 2016, CBuV has been reported in dogs in Canada, China, India, and Italy^20,23–25^. Although there are several reports of CBuVs in dogs with symptomatic and asymptomatic dogs, the pathogenesis of CBuV infection is still unclear. The previous studies suggested that CBuV infection may be associated with gastroenteritis symptoms in dogs^23,24^.

In Thailand, human bufavirus (HBuV) has been reported in patients with gastroenteritis and the environment^6,26^. The occurrence of Thai HBuV has been reported at 0.27% from diarrhea patients^6^. However, CBuV and its genetic characteristics have never been reported in dogs in Thailand. This study is the first to report the detection and characterization of canine bufavirus (CBuV), a novel parvovirus, in dogs in Thailand.

## Results

### Canine bufavirus (CBuV) in domestic dogs

From September 2016 to October 2022, we conducted a survey of canine bufavirus in domestic dogs. Rectal swabs were collected from domestic animals (n = 531) from 10 provinces of Thailand (Ayutthaya, Bangkok, Chiang Rai, Nakhon Ratchasima, Phayao, Ratchaburi, Samutprakarn, Samutsakorn, Suphanburi, and Tak) (Supplement Fig. 1). The samples were tested for CBuV using PCR with specific primers to the VP1/VP2 gene. CBuV was detected at 9.42% (50/531). CBuVs were found in both symptomatic (9.84%; 31/315) and asymptomatic dogs (8.80%; 19/216). By age group, CBuVs were detected in dogs of varying age groups, including those younger than 1 year (12.60%; 32/254) and dogs aged 1–5 years (7.34%; 13/177), with statistical significance (*p *< 0.05) (Supplement Table 1). CBuVs of positivity by month, the viruses could be detected every year from 2016 to 2022, and the highest occurrence of CBuVs was observed in November 2017 (100.00%). Regarding the positivity of CBuVs and seasons, the occurrence of CBuVs was highly detected during the winter season (November to January) and summer season (February to May). However, there was no statistically significant correlation between the positivity of CBuVs and seasons with *p* > 0.3288–1 (Supplement Table 2). The co-infection of CBuVs with other canine enteric viruses was observed, including CBuV/CPV-2 (n = 5), CBuV/CECoV (n = 11), CBuV/RVA (n = 2), CBuV/CPV-2/CECoV (n = 5), CBuV/CPV-2/CECoV/RVA (n = 1). Out of 50 CBuVs positive samples, 20 CBuVs were selected and sequenced for complete genome sequencing (n = 15) and partial VP2 gene (n = 5) (Table 1).

**Table 1:** Detailed description of Thai CBuVs characterized in this study.

Samples ID	Location	Collection date	Clinical signs	Age	Breed	Sequencing	Accession number
CU_FS53	Thailand	OCT/2016	Symptomatic	2 Months	Pomeranian	NS1, VP1, VP2	OQ730240
CU_FS70	Thailand	OCT/2016	Symptomatic	3 Months	Siberian Husky	Partial VP2	OQ730241
CU_FS231	Thailand	MAR/2017	Symptomatic	6 Months	Mixed	NS1, VP1, VP2	OQ730242
CU_FS232	Thailand	MAR/2017	Symptomatic	6 Months	Mixed	NS1, VP1, VP2	OQ730243
CU_FS235	Thailand	MAR/2017	Symptomatic	6 Months	Mixed	NS1, VP1, VP2	OQ730244
CU_FS236	Thailand	MAR/2017	Symptomatic	6 Months	Mixed	NS1, VP1, VP2	OQ730245
CU_FS20141	Thailand	NOV/2017	Symptomatic	3 Months	Chihuahua	NS1, VP1, VP2	OQ730246
CU_FS20932	Thailand	MAR/2018	Symptomatic	8 Months	Mixed	NS1, VP1, VP2	OQ730247
CU_FS22734	Thailand	NOV/2018	Symptomatic	3 Months	Mixed	NS1, VP1, VP2	OQ730248
CU_FS23266	Thailand	DEC/2018	Symptomatic	3 Months	Pomeranian	NS1, VP1, VP2	OQ730249
CU_FS23631	Thailand	FEB/2019	Symptomatic	3 Months	Mixed	Partial VP2	OQ730250
CU_FS26352	Thailand	JAN/2021	Symptomatic	3 Years	French bulldog	NS1, VP1, VP2	OQ730251
CU_FS26336	Thailand	JAN/2021	Asymptomatic	5 Years	French bulldog	Partial VP2	OQ730252
CU_FS26340	Thailand	JAN/2021	Asymptomatic	3 Years	French bulldog	NS1, VP1, VP2	OQ730253
CU_FS26359	Thailand	JAN/2021	Asymptomatic	3 Years	Pemborke Welsh Corgi	Partial VP2	OQ730254
CU_FS28678	Thailand	APR/2022	Symptomatic	5 Months	Pemborke Welsh Corgi	NS1, VP1, VP2	OQ730255
CU_FS28683	Thailand	APR/2022	Asymptomatic	8 Months	Pemborke Welsh Corgi	NS1, VP1, VP2	OQ730256
CU_FS28696	Thailand	APR/2022	Asymptomatic	5 Years	Pemborke Welsh Corgi	NS1, VP1, VP2	OQ730257
CU_FS28961	Thailand	MAY/2022	Symptomatic	6 Months	Mixed	NS1, VP1, VP2	OQ730258
CU_FS29327	Thailand	JUL/2022	Symptomatic	6 Years	Pomeranian	Partial VP2	OQ730259

### Phylogenetic and genetic analysis of CBuVs

In this study, we successfully sequenced the CBuVs, and the genome size of Thai CBuVs was 4214 bp. The genome structure of the virus contains non-structural protein (NS1) and viral capsid proteins (VP1 and VP2). Comparing the genome structure to other parvoviruses (canine parvovirus type-2 (CPV-2), canine bocavirus type 1 and 2(CBoV type 1 and 2), the genome structure of Thai CBuVs were similar to reference CBuV but diverse from CPV-2 and CBoV (Supplement Fig. 2).

**Figure 1:**
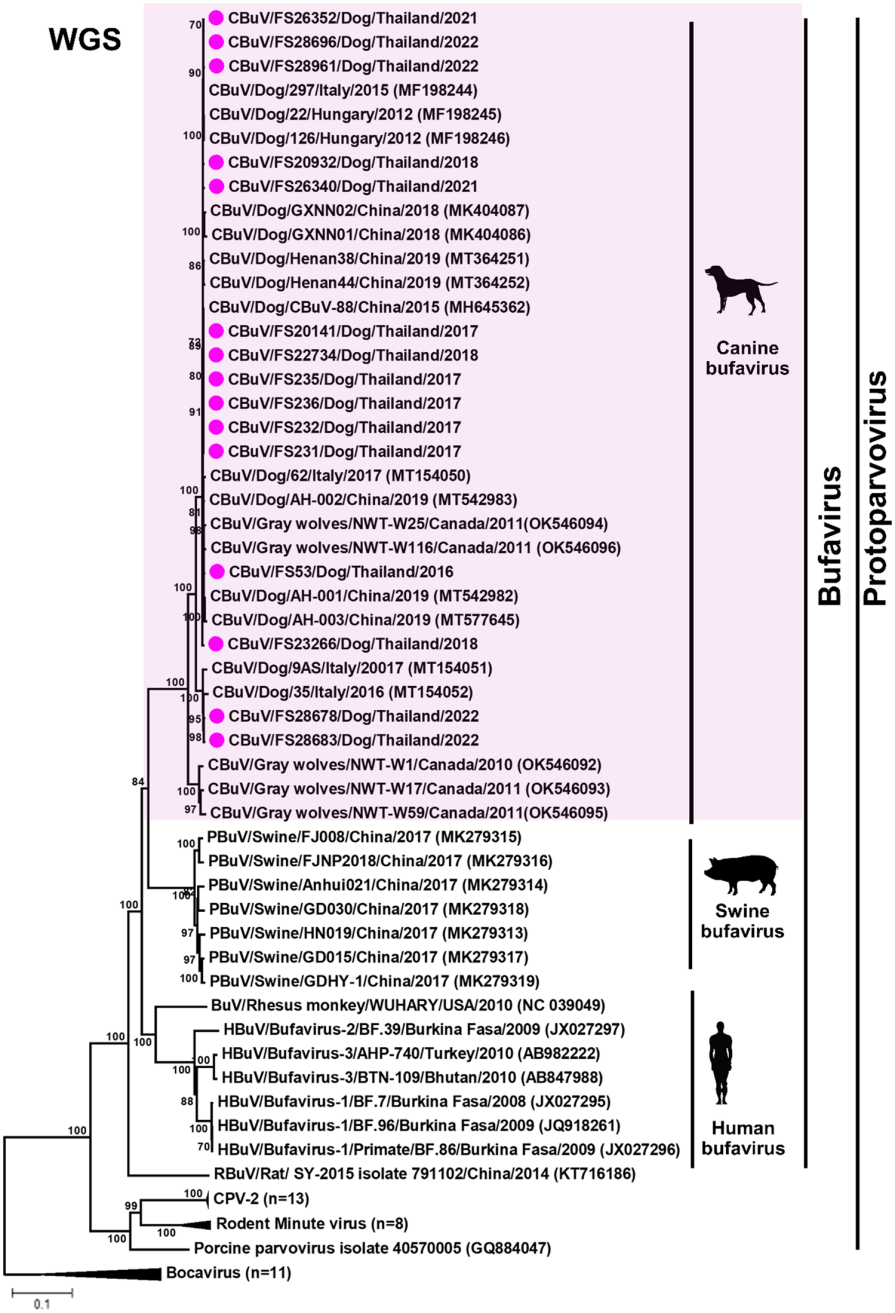
Phylogenetic analysis of the whole genome sequences of Thai CBuV. The phylogenetic tree was constructed using MEGA v.7.0 with a neighbor-joining algorithm with a Kimura-2 parameter model with 1000 replications of bootstrap analysis. The pink circle represents Thai CBuVs.

Phylogenetic analysis of the complete genome supported our observation that Thai CBuVs grouped with the canine CBuV group but in the separated cluster from BuVs from pigs, rats, and humans (Fig. 1). Pairwise comparison of the complete genome of CBuVs showed that Thai CBuVs possessed high nucleotide identity to the reference CBuVs from China and Italy with 95.20–99.70% nucleotide identities but low percentages of nucleotide identities with CPV-2 (57.90–58.00%) and CBoV (37.80–39.00%). The Thai CBuVs were closely related to Italy CBuV (ITA/2015/297, 99.70%), China CBuV (Henan38, 99.60%), and Hungary CBuV (HUN/2012/126, 99.60%) (Supplement Table 3). On the other hand, comparing Thai CBuVs and other bufaviruses from different hosts (humans, bats, rats, and pigs), the nucleotide identities ranged only from 43.20 to 65.20%.

**Figure 2:**
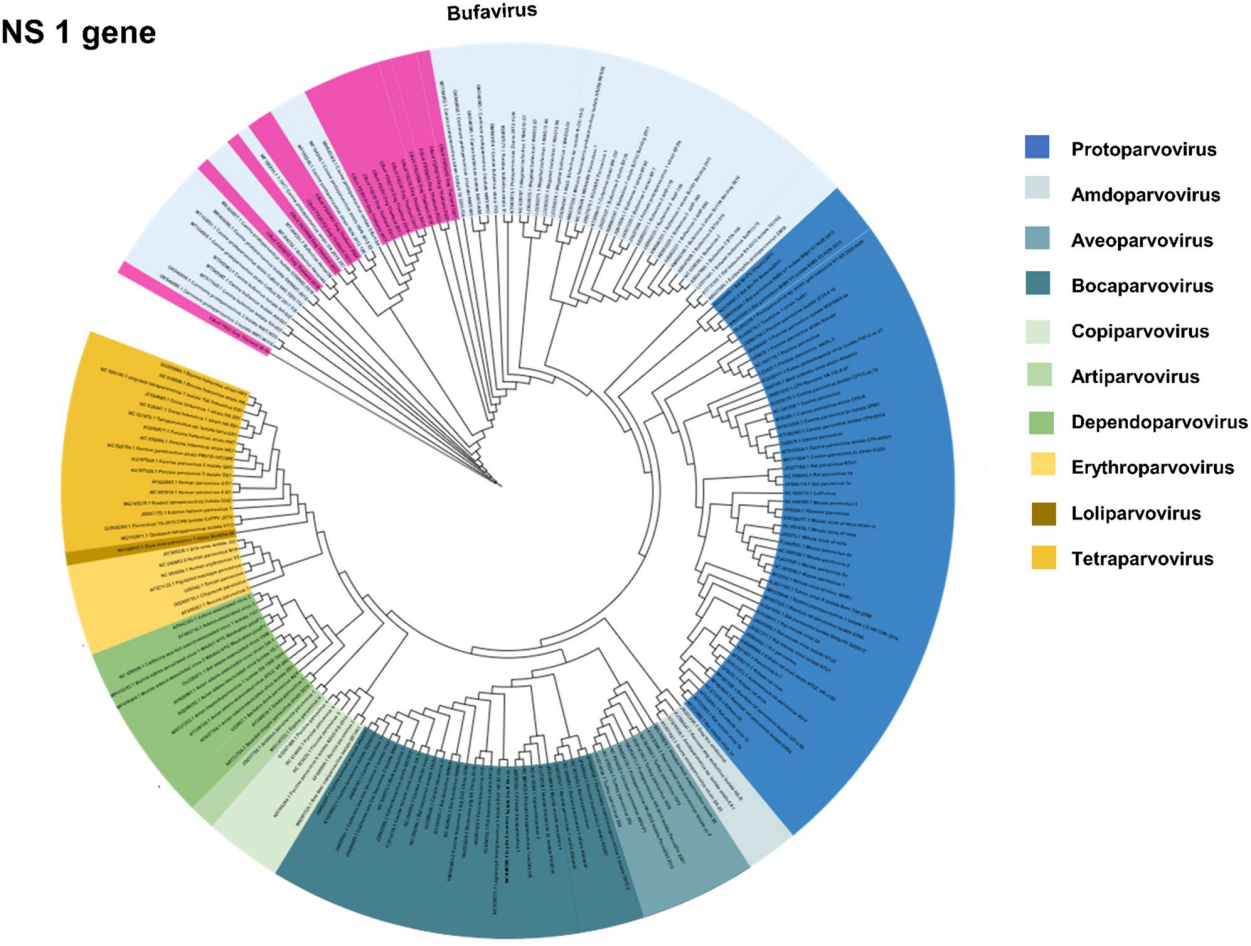
Phylogenetic analysis of the NS1 gene of Thai CBuV. The maximum likelihood tree was generated by using IQ-TREE version 2.1.3 (http://www.iqtree.org/) using the TVMe + IG4 model of nucleotide substitution, default heuristic search options, and ultrafast bootstrapping with 1000 replicates. The tree was visualized by iTOL version 6.0 (https://itol.embl.de/). The color indicated parvovirus genus including dark blue (Protoparvovirus), lite blue (Amdoparvovirus), teal (Aveoparvovirus), ocean (Bocaparvovirus), lite green (copiparvovirus), medium green (Artiparvovrisus), green (Depaendoparvovirus), lite yellow (Erythroparvovirus), dark yellow (Loliparvovirus), yellow (Tetraparvovirus). The pink color indicates Thai CBuVs in this study.

For NS1 gene, phylogenetic analysis of the NS1 gene showed that Thai CBuVs (n = 15) were grouped into BuVs of the Protoparvovirus genus with CBuVs from Canada, China, Hungary, and Italy but separated from other canine enteric parvoviruses of Protoparvovirus genus (CPV-2) and Bocaparovirus genus (CBoV) (Fig. 2). It is noted that based on NS1 phylogenetic analysis, *Parvovirinae* contains 10 genera including Amdoparvovirus, Aveoparvovirus, Artiparvovirus, Bocaparvovirus, Copiparvovirus, Dependoparvovirus, Erythroparvovirus, Loliparvovirus, Protoparvovirus and Tetraparvovirus. For NS1 nucleotide comparison, Thai CBuVs were highly conserved and possessed high nucleotide identities to Italy CBuV (ITA/2015/297; 99.90%), China CBuVs (CBuV-88; 99.80%) and Hungry CBuV (HUN/2012/126; 99.80%). Moreover, the Thai CBuVs possessed low nucleotide identities with bufaviruses of other species from bats, pigs, rats, and humans (62.50–68.40% nucleotide identities) (Supplement Table 3).

For VP1 and VP2 genes, based on phylogenetic analysis, BuVs can be divided into subgroups based on host species, e.g., human, canine, swine, and bat subgroups. The CBuVs can be further divided into 2 subgroups: A and B. The Thai CBuVs (n = 18) were grouped into subgroup A, which were closely related to CBuVs from China and Italy. While 2 Thai CBuVs (CU_FS 28678 and CU_FS 28683) were grouped into subgroup B, which were like Italy CBuVs (35/ITA and 9AS/ITA) (Supplement Fig. 3 and 4). VP1 and VP2 genes of Thai CBuVs possessed the highest nucleotide identities to those of reference CBuVs (98.70–99.80%). Thai CBuVs (subgroup A) possessed the highest nucleotide identities to China CBuVs (Henan38; 99.80%) but low percentages of nucleotide identities to CPV-2 (51.40–52.70%) and CBoV (40.60–43.30%). Thai CBuVs (subgroup B; CU_FS 28678 and CU_FS 28683) possessed the highest nucleotide identities to Italy CBuVs (35/ITA (98.7–99.2%) and 9AS/ITA (98.6–99.8%).

Genetic analysis of the NS1 gene showed that the NS1 gene of CBuVs contains 1917 nucleotides (639 amino acids). The conserved replication initiator motifs (GLHFHVLLQ and IVRYFLTKQP) were observed to be identical in all reference CBuVs but were not present in human bufavirus (GLHIHVLVC and IANYFLIKKP). The conserved amino acids at the walker loop ATP or GTP binding motifs of Thai CBuVs (GPASTGKS) were observed in both CBuVs and other bufaviruses (Table 2).

**Table 2:** Genetic analysis of Thai CBuV and reference viruses.

Virus	Species	Accession number	Gene						
			NS1			VP1			VP2
			Conserved replication initiator motifs		Walker loop [GXXXXGK(T/S)] ATP or GTP binding motifs	PLA2			
						Ca2 + binding loop (YXGXGYXGXR, YXGXF)	Catalytic center HDXXY	D	Glycine–rich sequence GGGGGGGSGVG
This study									
CU_FS53	Dog	OQ730240	GLHFHVLLQ	IVRYFLTKQP	GPASTGKS	YLGPG	HDLEY	D	GGGGGGGSGVG
CU_FS70	Dog	OQ730241	N/A	N/A	N/A	N/A	N/A	N/A	GGGGGGGSGVG
CU_FS231	Dog	OQ730242	GLHFHVLLQ	IVRYFLTKQP	GPASTGKS	YLGPG	HDLEY	D	GGGGGGGSGVG
CU_FS232	Dog	OQ730243	GLHFHVLLQ	IVRYFLTKQP	GPASTGKS	YLGPG	HDLEY	D	GGGGGGGSGVG
CU_FS235	Dog	OQ730244	GLHFHVLLQ	IVRYFLTKQP	GPASTGKS	YLGPG	HDLEY	D	GGGGGGGSGVG
CU_FS236	Dog	OQ730245	GLHFHVLLQ	IVRYFLTKQP	GPASTGKS	YLGPG	HDLEY	D	GGGGGGGSGVG
CU_FS20141	Dog	OQ730246	GLHFHVLLQ	IVRYFLTKQP	GPASTGKS	YLGPG	HDLEY	D	GGGGGGGSGVG
CU_FS20932	Dog	OQ730247	GLHFHVLLQ	IVRYFLTKQP	GPASTGKS	YLGPG	HDLEY	D	GGGGGGGSGVG
CU_FS22734	Dog	OQ730248	GLHFHVLLQ	IVRYFLTKQP	GPASTGKS	YLGPG	HDLEY	D	GGGGGGGSGVG
CU_FS23266	Dog	OQ730249	GLHFHVLLQ	IVRYFLTKQP	GPASTGKS	YLGPG	HDLEY	D	GGGGGGGSGVG
CU_FS26352	Dog	OQ730251	GLHFHVLLQ	IVRYFLTKQP	GPASTGKS	YLGPG	HDLEY	D	GGGGGGGSGVG
CU_FS26336	Dog	OQ730252	N/A	N/A	N/A	YLGPG	HDLEY	D	GGGGGGGSGVG
CU_FS26340	Dog	OQ730253	GLHFHVLLQ	IVRYFLTKQP	GPASTGKS	YLGPG	HDLEY	D	GGGGGGGSGVG
CU_FS26359	Dog	OQ730254	N/A	N/A	N/A	YLGPG	HDLEY	D	GGGGGGGSGVG
CU_FS28678	Dog	OQ730255	GLHFHVLLQ	IVRYFLTKQP	GPASTGKS	YLGPG	HDLEY	D	GGGGGGGSGVG
CU_FS28683	Dog	OQ730256	GLHFHVLLQ	IVRYFLTKQP	GPASTGKS	YLGPG	HDLEY	D	GGGGGGGSGVG
CU_FS28696	Dog	OQ730257	GLHFHVLLQ	IVRYFLTKQP	GPASTGKS	YLGPG	HDLEY	D	GGGGGGGSGVG
CU_FS28961	Dog	OQ730258	GLHFHVLLQ	IVRYFLTKQP	GPASTGKS	YLGPG	HDLEY	D	GGGGGGGSGVG
Reference									
ITA/2015/297	Dog	MF198244	GLHFHVLLQ	IVRYFLTKQP	GPASTGKS	YLGPG	HDLEY	D	GGGGGGGSGVG
HUN/2012/22	Dog	MF198245	GLHFHVLLQ	IVRYFLTKQP	GPASTGKS	YLGPG	HDLEY	D	GGGGGGGSGVG
CBuV-88	Dog	MH645362	GLHFHVLLQ	IVRYFLTKQP	GPASTGKS	YLGPG	HDLEY	D	GGGGGGGSGVG
GXNN02-2018	Dog	MK404087	GLHFHVLLQ	IVRYFLTKQP	GPASTGKS	YLGPG	HDLEY	D	GGGGGGGSGVG
CaBuV/62/2017	Dog	MT154050	GLHFHVLLQ	IVRYFLTKQP	GPASTGKS	YLGPG	HDLEY	D	GGGGGGGSGVG
HUN/2012/126	Dog	MF198246	GLHFHVLLQ	IVRYFLTKQP	GPASTGKS	YLGPG	HDLEY	D	GGGGGGGSGVG
CaBuV/9AS/2005/ITA	Dog	MT154051	GLHFHVLLQ	IVRYFLIKQP	GPASTGKS	YLGPG	HDLEY	D	GGGGGGGSGVG
CaBuV/35/2016/ITA	Dog	MT154052	GLHFHVLLQ	IVRYFLTKQP	GPASTGKS	YLGPG	HDLEY	D	GGGGGGGSGVV
AH-001	Dog	MT542982	GLHFHVLLQ	IIRYFLTKQP	GPASTGKS	YLGPG	HDLEY	D	GGGGGGGSGVG
AH-002	Dog	MT542983	GLHFHVLLQ	IVRYFLTKQP	GPASTGKS	YLGPG	HDLEY	D	GGGGGGGSGVG
AH-003	Dog	MT577645	GLHFHVLLQ	IIRYFLTKQP	GPASTGKS	YLGPG	HDLEY	D	GGGGGGGSGVG
Henan44	Dog	MT364252	GLHFHVLLQ	IVRYFLTKQP	GPASTGKS	YLGPG	HDLEY	D	GGGGGGGSGVG
Henan38	Dog	MT364251	GLHFHVLLQ	IVRYFLTKQP	GPASTGKS	YLGPG	HDLEY	D	GGGGGGGSGVG
NWT-W25	Dog	OK546094	GLHFHVLLQ	IVRYFLTKQP	GPASTGKS	YLGPG	HDLEY	D	GGGGGGGSGVG
NWT-W116	Dog	OK546096	GLHFHVLLQ	IVRYFLTKQP	GPASTGKS	YLGPG	HDLEY	D	GGGGGGGSGVG
Human and primate									
BF.7 NS1	BuV-1	JX027295	GLHIHVLVC	IANYFLIKKP	GPASTGKS	YLGPF	HDLEY	D	GGGGGGGSGVG
BF.96	BuV-1	JQ918261	GLHIHVLVC	IANYFLIKKP	GPASTGKS	YLGPF	HDLEY	D	GGGGGGGSGVG
BJ13	BuV-1	KM580347	GLHIHVLVC	IANYFLIKKP	GPASTGKS	YLGPF	HDLEY	D	GGGGGGGSGVG
BF.39	BuV-2	JX027297	GLHIHVLVC	IANYFLIKKP	GPASTGKS	YLGPF	HDLEY	D	GGGGGGGSGVG
BTN-109	BuV-3	AB847988	GLHIHVLVC	IANYFLIKKP	GPASTGKS	YLGPF	HDLEY	D	GGGGGGGSGVG
BTN-310	BuV-3	AB847989	GLHIHVLVC	IANYFLIKKP	GPASTGKS	YLGPF	HDLEY	D	GGGGGGGSGVG
AHP-740	BuV-3	AB982222	GLHIHVLVC	IANYFLIKKP	GPASTGKS	YLGPF	HDLEY	D	GGGGGGGSGIG
BF.86	Primate	JX027296	GLHIHVLVC	IANYFLIKKP	GPASTGKS	YLGPF	HDLEY	D	GGGGGGGSGVG
Bat									
ZM38	Bat	AB937988	GLHIHLLLQ	IFNYFLTKEI	GPASTGKS	YLGPG	HDLAY	D	GGGGGGGSGVG
Bat Mr-PV	Bat	KC154060	GLHFHVLLQ	ICHYFLKKQI	GPASTGKS	YLGPG	HDLEY	D	GGGGGGGSGVG
Bat Ms-PV	Bat	KC154061	GLHFHVLLQ	IVRYFLTKQP	GPASTGKS	YLGPG	HDLEY	D	GGGGGGGSGVG
BtBV/V7/HUN/2013	Bat	KR078344	GLHFHVLLQ	IVRYFLTKQP	GPASTGKS	YLGPG	HDLEY	D	GGGGGGGSGVG
MAG12-57	Bat	LC085675	GLHFHVLLQ	IVRYFLTKQP	GPASTGKS	YLGPG	HDLEY	D	GVGGAGGGGVG
Porcine									
GD015	Porcine	MK279317	N/A	N/A	N/A	YLGPG	HDLEY	D	GVSGGAGGGGVG
GD030	Porcine	MK279318	N/A	N/A	N/A	YLGPG	HDLEY	D	GVSGGAGGGGVG
GDHY-1	Porcine	MK279319	N/A	N/A	N/A	YLGPG	HDLEY	D	GASGGGGGGGVG
SY-2015	Rat	NC 028650	N/A	N/A	N/A	YLGPG	HDLAY	D	GGGGGGGSGVG

VP1 gene of CBuVs contains 2130 nucleotides (710 amino acids). Thai CBuVs contained three potential sites for PLA2 activity, which is required for viral entry. At one site, the calcium binding loop, Thai CBuVs contained amino acid residues (YLGPG) similar to other bufaviruses from dogs, bats, and pigs but were not present in human bufaviruses (YLGPF). The other, two catalytic sites contain amino acid residues (HDLEY and D) similar to all reference BuVs (Table 2). Interestingly, amino acid residues of VP1 related to host preference (human specific) were observed in Thai CBuVs, including PTNRP3-6AIRKA, G22F, T24Q, N71D, K86R, and K89R. This observation could suggest preference characteristics of BuVs to human hosts (Table 3).

VP2 gene of CBuVs contains 1704 nucleotides (568 amino acids). A glycine-rich motif (G-rich) was observed at the N-terminus of VP1, which was similar to other parvoviruses (Table 2). This motif is speculated to be associated with the cellular entry of the virus. Thai CBuVs subgroup B (CU_FS28678 and CU_FS28683) contained 16 unique amino acid residues, which were similar to CBuVs strain 9AS and 35 from Italy, suggesting unique subgroup B characteristics (Supplement Table 4). Moreover, amino acid residues related to host preference were observed. Amino acid insertion at 12–13 and 370 were observed in human BuVs and bat BuVs (Table 3).

**Table 3:** Genetic analysis of the primate and human-specific residues of Thai CBuV and reference BuVs.

Virus	Species	Accession number	Gene								
			VP1							VP2	
			3–6 PTNRP	9	22	24	71	86	89	12–13	370
This study											
CU_FS53	Dog	OQ730240	PTNRP	K	G	T	N	K	K	Deletion	Deletion
CU_FS231	Dog	OQ730242	PTNRP	K	G	T	N	K	K	Deletion	Deletion
CU_FS232	Dog	OQ730243	PTNRP	K	G	T	N	K	K	Deletion	Deletion
CU_FS235	Dog	OQ730244	PTNRP	K	G	T	N	K	K	Deletion	Deletion
CU_FS236	Dog	OQ730245	PTNRP	K	G	T	N	K	K	Deletion	Deletion
CU_FS20141	Dog	OQ730246	PTNRP	K	G	T	N	K	K	Deletion	Deletion
CU_FS20932	Dog	OQ730247	PTNRP	K	G	T	N	K	K	Deletion	Deletion
CU_FS22734	Dog	OQ730248	PTNRP	K	G	T	N	K	K	Deletion	Deletion
CU_FS23266	Dog	OQ730249	PTNRP	K	G	T	N	K	K	Deletion	Deletion
CU_FS26352	Dog	OQ730251	PTNRP	K	G	T	N	K	K	Deletion	Deletion
CU_FS26336	Dog	OQ730252	PTNRP	K	G	T	N	K	K	Deletion	Deletion
CU_FS26340	Dog	OQ730253	PTNRP	K	G	T	N	K	K	Deletion	Deletion
CU_FS26359	Dog	OQ730254	PTNRP	K	G	T	N	K	K	Deletion	Deletion
CU_FS28678	Dog	OQ730255	PTNRP	K	G	T	N	K	K	Deletion	Deletion
CU_FS28683	Dog	OQ730256	PTNRP	K	G	T	N	K	K	Deletion	Deletion
CU_FS28696	Dog	OQ730257	PTNRP	K	G	T	N	K	K	Deletion	Deletion
CU_FS28961	Dog	OQ730258	PTNRP	K	G	T	N	K	K	Deletion	Deletion
Reference											
ITA/2015/297	Dog	MF198244	PTNRP	K	G	T	N	K	K	Deletion	Deletion
HUN/2012/22	Dog	MF198245	PTNRP	K	G	T	N	K	K	Deletion	Deletion
CBuV-88	Dog	MH645362	PTNRP	K	G	T	N	K	K	Deletion	Deletion
GXNN02-2018	Dog	MK404087	PTNRP	K	G	T	N	K	K	Deletion	Deletion
CaBuV/62/2017	Dog	MT154050	PTNRP	K	G	T	N	K	K	Deletion	Deletion
HUN/2012/126	Dog	MF198246	PTNRP	K	G	T	N	K	K	Deletion	Deletion
CaBuV/9AS/2005/ITA	Dog	MT154051	PTNRP	K	G	T	N	K	K	Deletion	Deletion
CaBuV/35/2016/ITA	Dog	MT154052	PTNRP	K	G	T	N	K	K	Deletion	Deletion
AH-001	Dog	MT542982	PTNRP	K	G	T	N	K	K	Deletion	Deletion
AH-002	Dog	MT542983	PTNRP	K	G	T	N	K	K	Deletion	Deletion
AH-003	Dog	MT577645	PTNRP	K	G	T	N	K	K	Deletion	Deletion
Henan44	Dog	MT364252	PTNRP	K	G	T	N	K	K	Deletion	Deletion
Henan38	Dog	MT364251	PTNRP	K	G	T	N	K	K	Deletion	Deletion
NWT-W25	Wolf	OK546094	PTNRP	K	G	T	N	K	K	Deletion	Deletion
NWT-W116	wolf	OK546096	PTNRP	K	G	T	N	K	K	Deletion	Deletion
Human and non-human primate											
BF.7 NS1	Bufavirus-1	JX027295	AIRKA	Deletion	F	Q	D	R	R	SD	S
BF.96	Bufavirus-1	JQ918261	AIRKA	Deletion	F	Q	D	R	R	SD	S
BJ133/BeiJing/2011	Bufavirus-1	KM580347	AIRKA	Deletion	F	Q	D	R	R	SD	S
BF.39	Bufavirus-2	JX027297	AIRKA	Deletion	F	Q	D	R	R	PD	G
BTN-109	Bufavirus-3	AB847988	AIRKA	Deletion	F	Q	D	R	R	AE	G
BTN-310	Bufavirus-3	AB847989	AIRKA	Deletion	F	Q	D	R	R	AE	G
AHP-740	Bufavirus-3	AB982222	AIRKA	Deletion	F	Q	D	R	R	TE	G
BF.86	Primate	JX027296	AIRKA	Deletion	F	Q	D	R	R	SD	S
Bat											
Bat Mr-PV	Bat	KC154060	PTNRH	G	G	T	N	K	K	DA	G
Bat Ms-PV	Bat	KC154061	PTNRH	Q	G	T	N	K	K	DA	G
Porcine											
GD015	Porcine	MK279317	PTNRP	K	G	T	N	K	K	ND	G
GD030	Porcine	MK279318	PTNRP	K	G	T	N	K	K	ND	G
GDHY-1	Porcine	MK279319	PTNRP	K	G	T	N	K	K	ND	G

### Antigenic epitopes prediction, selective pressure, and recombination event of CBuVs

Based on VP2, the recommendation residues of antigenic epitopes prediction of Thai CBuV subgroup A (CU_FS20141) were at position 239–258 (KFDDIQFITVENCVPIELLR) and 99–118 (NDSYHAKVETPWSLLHANCW) which similar to CBuV strain AH001 and AH002 from China (MT542982, and MT 542983). While the residues of antigenic epitopes of Thai CBuV subgroup B (CU_FS28678) were at position 90–109 (QTLHGRDTINDSYHAKVETP) and 239–258 (KYDDIQFITVENCVPIELLR) (Supplement Table 5).

The selective pressure of CBuVs was analyzed by the alignment of all genes of CBuVs using the statistical parameters (FEL, FUBAR, and MEME). The potential positive selection sites of NS1 (n = 9), VP1 (n = 8), and VP2 (n = 7) were detected in this study (Supplement Table 6). The overall mean difference of dN/dS was 0.306 for the NS1 gene, 0.125 for the VP1 gene, and 0.118 for the VP2 gene, suggesting all gene of CBuVs was under negative selection. For the NS1 gene, only one amino acid at position 512 of NS1 was confirmed to be a positive selection (*p* < 0.1 by MEME and posterior probability of 0.9 by FUBAR). For the VP1 gene, the amino acid position at 256 was confirmed to be a positive selection site (*p* < 0.1 by MEME and FEL, posterior probability of 0.9 by FUBAR). For the VP2 gene, amino acid positions at 22 and 113 were found as positive selection (*p* < 0.1 by FEL and posterior probability of 0.9 by FUBAR). A potential positive selection at 113 of the VP2 gene located at the predicted B-cell epitopes of Thai CBuV (strain CU_FS20141) at the location 99–118.

**Figure 3:**
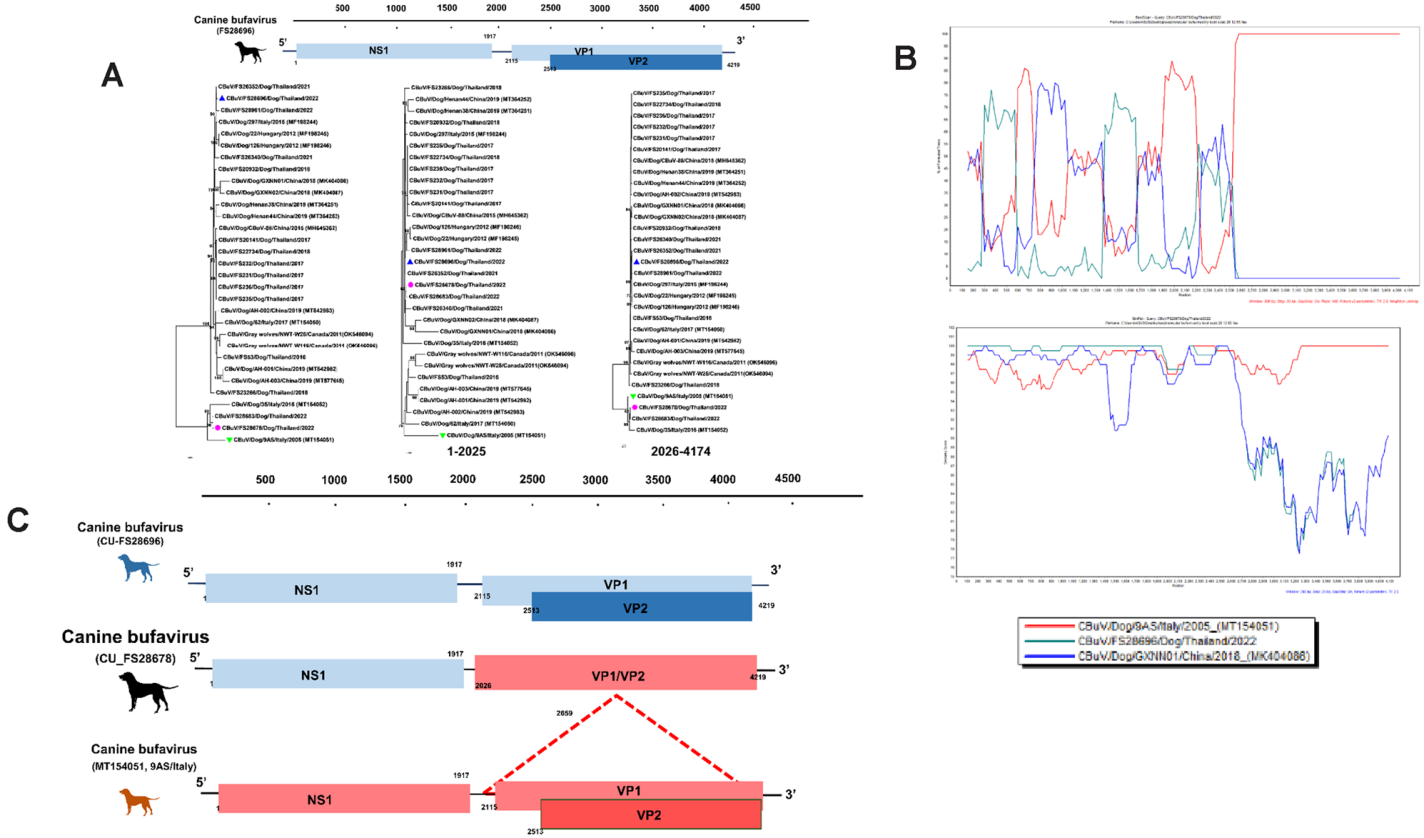
Phylogenetic analysis and recombination analysis of Thai CBuV (CU_FS 28678). (**a**) Phylogenetic analysis of CBuVs was constructed using MEGA v.7.0 with a neighbor-joining algorithm with a Kimura-2 parameter model with 1000 replications of bootstrap analysis. The pink circle represents Thai CBuV strain CU_FS 28678. The blue and green triangles showed putative major and minor parents. (**b**) Similarity and Bootscan analysis of Thai CBuV showed the recombinant CU_FS 28678. (**c**) Genome organization of potential recombinant of Thai CBuV strain CU_ FS 28678.

Recombinant analysis of Thai CBuV was performed by using the RDP program, similarity plot, and bootScan analysis. A putative recombinant event was observed in Thai CBuV (CU_FS28678) (Fig. 3). The putative recombinant breakpoint located at the position 1684–2697 which supported by statistically significant (RDP, GENECONV, BootScan, Maxchi, Chimaera, SiScan and 3Seq with *p*-value of 1.067 × 10^−07^, 5.806 × 10^−06^, 4.323 × 10^−05^, 1.144 × 10^−09^, 8.612 × 10^−03^, 7.014 × 10^−30^, 2.222 × 10^−21^, respectively) (Supplement Table 7). Our result showed that the major and minor putative parents of Thai CBuV (CU_FS28678) were CBuV strain 9AS from Italy (MT154051; CBuV-subgroup B) and Thai CBuV (CU_FS28696) (CBuV-subgroup A).

## Discussion

Bufavirus (BuV) is a novel member of the family *Parvoviridae*. It was first reported in humans with gastroenteric symptoms in 2012^8^. Bufavirus infection in dogs was first described in Italy in 2016 and subsequently reported in several countries^20,23,24,27^. However, CBuVs have not been reported in Thailand. This study is the first to detect CBuVs in domestic dogs in Thailand. Our survey showed CBuV positivity at 9.42% (50/531), which was comparable to other previous studies (2.5–8.8% positivity)^20,22–24^. However, there was no significant difference in CBuV positivity by season. Our result showed that CBuVs was highly detected in gastroenteritis dogs, although there was no statistical significance between symptomatic and asymptomatic dogs. The previous studies showed that the CBuVs have been detected in both healthy dogs and dogs with respiratory and gastroenteric symptoms^20,24,27^. Some studies supported that BuVs may associated with gastroenteritis and cause systemic infection in humans and dogs^10,11,23,24^. However, the pathogenesis of CBuV infection is still not clear. Thai CBuVs showed a significant presence in both younger than 1 year (< 1 year) and dogs aged 1–5 years with statistical significance (*p* < 0.05) (Supplement Table 1). This observation agreed with the previous study, which suggested that CBuV tends to be more prevalent for dogs under 5 years of age than the older age group^20^. In this study, we observed co-infection of CBuVs with other enteric viruses. For example, CPV-2 and CECoV were co-infection with CBuV, which was similar to other studies^23,27^. However, the severity level of clinical signs of co-infection between CBuVs and other pathogens should be further investigated.

Currently, only 17 nearly complete genome sequences of CBuVs are available in the GenBank Database. Our study provided an additional 15 complete genomes of CBuVs in the database. In this study, the complete genome size of CBuVs encoding 3 ORFs, including NS1, VP1, and VP2 (Supplement Fig. 2). Based on the phylogenetic analysis of the complete genome, Thai CBuVs belong to the bufavirus of the Protoparvovirus genus (canine group) and were closely related to CBuVs from China (Henan38), Hungary (HUN/2012/126) and Italy (ITA/2015/297). It is noted that the classification of parvovirus genus was classified by NS1 gene sequencing^28^. The phylogenetic analysis of the NS1 gene showed that Thai CBuVs were grouped into the Protoparvovirus genus (CBuV group) but were separated from canine parvovirus type -2 (CPV-2). Thai CBuVs have high nucleotide identities to reference CBuVs at 98.70–99.90% but were distinct from CPV-2 viruses at only 57.10% nucleotide identities. The phylogenetic analysis of VP1 and VP2 genes showed that CBuVs clustered into 2 major subgroups, subgroups A and B, which agreed with the previous study^23^. Thai CBuVs (n = 18) were grouped into subgroup A with CBuVs from Italy and China. The other Thai CBuVs (strain CU_FS 28678 and CU_ FS 28683; n = 2) were grouped into subgroup B with CBuV from Italy. It is noted that Thai CBuVs (subgroups A and B) might share the same common ancestor with CBuVs from China and Italy.

Thai CBuVs contained conserved regions of the parvovirus-conserved replication initiator motifs. For NS1, Thai CBuVs contained a helicase motif walker (GPASTGKS), which was also observed in all reference bufaviruses^8,20^. For the VP1 gene, Thai CBuVs posed a unique calcium-binding loop site (YLGPG) and two catalytic sites (HDLEY and D), which were also observed in most reference bufaviruses, suggesting unique characteristics^11,18^. Previous studies indicated that the calcium-binding loop site and phospholipase A2 (PLA2) region may be associated with viral entry to the host cell of the viruses^29^. The N-terminus of the VP1/VP2 had a glycine-rich motif, which may be associated with the cellular entry of parvovirus^30,31^. Moreover, human-specific amino acid residues of VP1 were observed at 3-6AIRKA, 22F, 24Q, 71D, 86R, and 89R. Unique amino acid insertion at positions 12–13 and 370 of VP2 were observed in human BuVs and Bat BuVs, suggesting potential antigenic markers for human and bat BuVs. Moreover, in the comparison of the genetic analysis between CBuVs subgroup A and B, there were 16 unique amino acids of CBuVs subgroup B, suggesting the genetic characteristics of the CBuV subgroup.

Our study showed that the VP1 and VP2 genes of Thai CBuVs have higher variations than the NS1 gene, which were similar to the previous study^32^. The VP2 gene of parvoviruses may have functions relating to viral entering, receptor binding, and immunogenicity and contains many major epitopes^32–34^. Moreover, B-cell epitopes are recognized as an immunogenicity classification. Thus, the mutations of this epitope may affect immunogenicity or generate a novel serotype of the virus^35^. The top list of antigenic epitope predictions on the VP2 gene of Thai CBuVs (CU_FS 20141 and CU_FS 28678) were observed in this study at 239–258 and 90–109, respectively (Supplement Table 5). In the selective pressure analysis based on the VP2 gene, two positive selections (22, 113) were observed in this study. A residue at 113 of VP2 was located at predicted B-cell epitopes of Thai CBuV (CU_FS20141) (location 99–118). Notably, the possibility of an antigenic shift to escape host immune responses of Thai CBuVs should be considered. However, due to the limited number of isolates in this study, further investigation on an extensive sample scale is necessary.

The positive selection pressure and genetic recombination are the factors that affect to high evolution rate of parvovirus evolution^36,37^. Previous study showed that natural recombination events of protoparvovirus between bufaviruses strains WUHARV and MgBuV1 can occur, suggesting cross-species transmission or sharing a common ancestor between bat, swine, and non-human primate bufavirus^15^. In this study, the recombinant Thai CBuV (CU_FS28678) was observed between Thai CBuV (CU_FS28696) and Italy CBuVs (9AS), suggesting a possible common ancestor of Thai and Italy CBuVs and inducing genetic diversity of CBuVs.

In conclusion, this is the first to report and genetically characterize the complete genome of CBuVs in domestic dogs in Thailand. Our result showed that Thai CBuVs were detected in both healthy and dogs with gastroenteric signs. The phylogenetic analysis showed that Thai CBuVs might share a common ancestor with CBuVs from Italy and China. However, the genetic database of CBuVs is still limited. Thus, surveillance and genetic characterization of CBuVs in domestic animals should be further investigated on a larger scale to elucidate the dynamic, evolution, and distribution of CBuVs.

## Materials and methods

### Canine samples

In this study, we conducted a cross-sectional sample collection from Chulalongkorn University’s Veterinary Teaching Hospital and private small animal hospitals in Thailand from September 2016 to October 2022. A total of 531 rectal swab samples were collected from dogs with asymptomatic (n = 216) and gastroenteritis (n = 315) symptoms, including vomiting, diarrhea, and dehydration. The samples were collected from dogs of young age (< 1 year) (n = 254), adults (1–5 years) (n = 177), and seniors (> 5 years) (n = 100). The animal demographic data, including age, sex, breed, and vaccination history, were recorded. This study was performed in accordance with the Chulalongkorn University, Animal Care and Uses Protocol (CU-VET IACUC#2031050, CU-VET IACUC#2031035) guidelines and regulations.

### Canine bufavirus identification

All rectal swab samples were subjected to DNA extraction using DNA/RNA GENTi Automated Nucleic Acid Extraction System (GeneAll® Seoul, Korea) following the manufacturer’s recommendations. For canine bufavirus identification, DNA samples were screened using PCR with specific primers for the VP1/VP2 gene. The primers used in this study were previously described, including CPPV 165F (5′-CTGGTTTAATCCAGCAGACT3′), CPPV 371R (5′-TGAAGACCAAGGTAGTAGGT3′) corresponded to the position 2872–2891 and 3060–3079 of CBuV, respectively^22^. The PCR was performed in a final volume of 50 μl comprising 4 μl of template DNA, 25 μl of 2 × reaction buffer of the HotStarTaq® Master Mix (Qiagen, Hilden, Germany), 0.2 μM of each forward and reverse primers and distilled water to a final volume of 50 μl. The PCR condition included an initial denaturation step at 95 °C for 15 min, following 40 cycles of denaturation at 94 °C for 30 s, annealing at 52 °C for 30 s, and extension at 72 °C for 1 min, as well as a final extension step at 72 °C for 10 min. PCR products were run on a 1.5% agarose gel, which was mixed with RedSafe™ (iNtRON Biotechnology, Inc., Korea) at 100 V for 45 min. The expected size of the CBuV-positive sample was 208 bp. In this study, other canine viral enteric pathogens, including Canine Parvovirus, Canine Coronavirus, and Canine Rotavirus, were also tested in all samples^38–40^.

### Canine bufavirus characterization

Representatives of positive CBuV were selected for whole genome sequencing (n = 15) and VP1/VP2 sequencing (n = 5). The CBuVs were selected based on epidemiological and demographic data such as age, collection date, breed, and vaccination history. Whole genome sequencing was conducted by amplification of each gene of the viruses by using modified oligonucleotide primer sets as previously described and new primer sets designed by using the Primer 3 plus program (https://www.bioinformatics.nl/cgi-bin/primer3plus/primer3plus.cgi) (Supplement Table 8)^22,23,25,41^. Nucleotide amplification was conducted in 50 μl PCR reaction comprising 4 μl of template DNA, 25 μl of 2 × reaction buffer of the HotStarTaq® Master Mix (Qiagen, Hilden, Germany), 0.2 μM of each forward and reverse primer and distilled water to a final volume of 50 μl. The PCR condition was set as initial denaturation step at 95 °C for 15 min; 40 cycles of denaturation at 94 °C for 30 s, annealing at 50–55 °C for 30 s and extension at 72 °C for 1–1.30 min, and final extension step at 72 °C for 10 min. PCR products were then purified and sequenced at 1st Base Laboratories Sdn Bhd, Malaysia. The nucleotide sequences were edited, validated, and assembled using SeqMan software v.5.03 (DNASTAR Inc.; Wisconsin, USA). In this study, whole genome and VP2 sequences of Thai CBuVs were deposited into the GenBank database under the accession numbers OQ730240- OQ730259 (Table 1).

### Phylogenetic and genetic analyses of canine bufavirus

Phylogenetic and genetic analyses of CBuV were conducted by comparing nucleotide sequences of Thai CBuVs with those of reference viruses of *the Parvoviridae* family available from the GenBank database, including canine parvovirus, canine bocavirus, human bufavirus, bat bufavirus, rat bufavirus, and swine bufavirus. The reference nucleotide sequences of CBuV were also included. It should be noted that only 17 complete genome sequences of domestic dog and canid wildlife BuVs have been reported and were available from the GenBank database. Phylogenetic trees of WGS and VP1 gene of CBuVs were constructed using MEGA v.7.0 (Tempe, AZ, USA) with the neighbor-joining method applied with the Kimura 2-parameter and 1000 bootstrap replicates. For NS1 and VP2, phylogenetic tree and best models were generated by using IQ-TREE version 2.1.3 (http://www.iqtree.org/) with the TVMe + IG4 model of nucleotide substitution, default heuristic search options, and ultrafast bootstrapping with 1000 replicates. The tree was visualized by iTOL version 6.0 (https://itol.embl.de/) and Figtree V1.3.1 (http://tree.bio.ed.ac.uk › software).

For genetic analysis, the nucleotide sequences and deduced amino acids of CBuVs were aligned and compared with those of reference viruses using MegAlign software v.5.03 (DNASTAR Inc.; Wisconsin, USA). A pairwise comparison of nucleotides and amino acids of Thai CBuV, and those of reference CBuV was conducted. The variable and unique amino acids related to receptor binding of the viruses and host preferences were evaluated. For analysis of the selective pressure of CBuVs, the ratio of non-synonymous (dN) to synonymous (dS) substitutions was estimated using static methods on the online software (http://www.datamonkey.org/). The values dN/dS > 1, dN/dS = 1, and dN/dS < 1 were used to define positive selection, neutral mutations, and negative selection, respectively. The positive selection position site was identified by at least 2 algorithms. The significance levels were set at *p* = 0.1. Antigenic epitope prediction of Thai CBuVs was identified using the online software (http://sysbio.unl.edu/SVMTRiP/). Recombination analysis was performed using the Recombination Detection Program (RDP) package version 4.0 with a statistical method including RDP, GENECONV, BootScan, MaxChi, Chimaera, SiScan, and 3Seq. The potentially positive recombination was analyzed using a potential breakpoint signal of at least four methods with *p*-values < 0.01. The related phylogenetic tree with potential recombinant and its putative major and minor parents were generated using RDP 4 package software.

### Statistical analysis

Correlation among CBuVs positivity and the sample collection date, age of dogs, and clinical signs were analyzed using Fisher's exact test (https://www.socscistatistics.com/tests/fisher). A *p*-value of < 0.05 was considered as statistical significance.

### Ethics statement

This study was conducted under the approval of the Institute for Animal Care and Use Protocol of the CU-VET, Chulalongkorn University (CU-VET IACUC#2031050, CU-VET IACUC#2031035).

### Supplementary Information

Supplementary Information.
