# Supplementary material for: First detection and genetic characterization of canine bufavirus in domestic dogs, Thailand
Source: Sci Rep. 2024 Feb 27;14:4773. doi: 10.1038/s41598-024-54914-z (PMC10899236; doi:10.1038/s41598-024-54914-z)
Supplement: Supplementary file 1 — Supplementary Information. [file 41598_2024_54914_MOESM1_ESM.docx]

**Supplementary Material**

**First detection and genetic characterization of canine bufavirus in domestic dogs, Thailand**

Kamonpan Charoenkul^1,2^, Yu Nandi Thaw^2^, Eaint Min Phyu^2^, Waleemas Jairak^1,2^, Chanakarn Nasamra^1,2^, Ekkapat Chamsai^1^, Supassama Chaiyawong^1,2^,

and Alongkorn Amonsin^1,2*^

^1^ Center of Excellence for Emerging and Re-emerging Infectious Diseases in Animals, Faculty of Veterinary Science, Chulalongkorn University, Bangkok, Thailand

^2^ Department of Veterinary Public Health, Faculty of Veterinary Science, Chulalongkorn University, Bangkok, Thailand

*Corresponding author: Professor Dr. Alongkorn Amonsin

Mailing address: Department of Veterinary Public Health, Faculty of Veterinary Science, Chulalongkorn University, Bangkok, Thailand 10330

Phone: +66 2218 9578 Fax: +66 2218 9577

E-mail: Alongkorn.a@chula.ac.th

**Keywords: Canine Bufavirus, Characterization, Dog, Genetic, Thailand**

Running Head:

Characterization of CBuV in Thailand

**Supplement Tables**

Supplement Table 1. Percentage of positive CBuVs by clinical signs and age groups

Supplement Table 2. Percentage of positive CBuVs by months and seasons

Supplement Table 3. Pairwise comparison of whole genome sequences of Thai CBuV (CU_FS231) with reference parvoviruses

Supplement Table 4. Genetic analysis of VP2 gene between Thai CBuVs and reference CBuVs.

Supplement Table 5. Prediction of antigenic epitopes of Thai CBuV strain CU_FS20141 and CU_FS28678

Supplement Table 6. Evidence for positive and negative selection using various detection methods for CBuVs

Supplement Table 7. Recombinant analysis: CBuV strain CU_FS28678

Supplement Table 8. Nucleotide sequences of primers used for CBuV detection and sequencing in this study.

**Supplement Figure**

Supplement Figure 1 Location of sample collection and CBuVs detected by provinces in this study (Map of Thailand with permission by World Trade Press)

Supplement Figure 2 Genome structure of Canine parvoviruses and BuVs

Supplement Figure 3 Phylogenetic analysis of VP1 gene of Thai CBuV, the phylogenetic tree was constructed by using MEGA v.7.0 with neighbor-joining algorithm with Kimura-2 parameter model with 1,000 replications of bootstrap analysis. The pink circle represents Thai CBuVs.

Supplement Figure 4 Phylogenetic analysis of VP2 gene of Thai CBuV, the maximum likelihood tree constructed by using IQ-TREE version 2.1.3 (http://www.iqtree.org/) using the TVMe +IG4 model of nucleotide substitution, default heuristic search options, and ultrafast bootstrapping with 1000 replicates. Tree was visualized by iTOL version 6.0 (<https://itol.embl.de/>). The pink circles indicate the Thai CBuVs.

**Supplement Table 1.** Percentage of positive CBuVs by clinical signs and age groups

| **Year** | **Percentage of positive CBuVs** | | | | | |
| --- | --- | --- | --- | --- | --- | --- |
|  | **Clinical signs (%)** | | **Age groups (%)** | | |  |
|  | **Healthy** | **Symptomatic** | **< 1 year** | **1-5 years** | **>5 years** |  |
| **2016** | 1/22 (4.55) | 4/40 (10.00) | 2/26 (7.69) | 1/27 (3.70) | 2/9 (22.22) |  |
| **2017** | 0/22 (0.00) | 16/91 (17.8) | 16/77 (20.78) | 0/23 (0.00) | 0/13 (0.00) |  |
| **2018** | 0/2 (0.00) | 4/88 (4.55) | 4/47 (8.51) | 0/20 (0.00) | 0/23 (0.00) |  |
| **2019** | 0/0 (0.00) | 3/67 (4.48) | 3/47 (6.38) | 0/16 (0.00) | 0/4 (0.00) |  |
| **2020** | 1/53 (1.89) | 0/0 (0.00) | 0/28 (0.00) | 0/12 (0.00) | 1/13 (7.69) |  |
| **2021** | 9/50 (18.00) | 0/4 (0.00) | 0/9 (0.00) | 8/30 (26.67) | 1/15 (6.67) |  |
| **2022** | 8/67 (11.94) | 4/25 (16.00) | 7/20 (33.33) | 4/49 (8.16) | 1/23 (4.35) |  |
| **Total** | 19/216 (8.80) | 31/315 (9.84) | 32/254 (12.60) | 13/177 (7.34) | 5/100 (5.00) |  |

- Fisher exact test; p=0.7629 (p <0.05) not statistical significance when compared to the healthy and symptomatic.
- Fisher exact test; p= 1 (p <0.05) not statistical significance when compared to < 1 year and 1-5 years
- Fisher exact test; p= 0.0196 (p <0.05) statistical significance when compared to < 1 year and >5 years
- Fisher exact test; p= 0.0345 (p <0.05) statistical significance when compared to 1-5 year and >5 years

**Supplement Table 2.** Percentage of positive CBuVs by months and seasons

| 1. **Percentage of positive CBuVs by months** | | | |
| --- | --- | --- | --- |
| **Year** | **Month** | **Total samples** | **Number positive for CBuVs (%)** |
| 2016 | Sep | 7 | 3 (42.86) |
|  | Oct | 25 | 2 (8.00) |
|  | Nov | 12 | 0 |
|  | Dec | 18 | 0 |
| 2017 | Jan | 17 | 2 (11.76) |
|  | Feb | 22 | 2 (9.09) |
|  | Mar | 6 | 4 (66.67) |
|  | Apr | 7 | 0 |
|  | May | 3 | 1 (33.33) |
|  | June | 12 | 1 (8.33) |
|  | July | 10 | 1 (10.00) |
|  | Aug | 4 | 0 |
|  | Sep | 15 | 1 (6.67) |
|  | Oct | 4 | 0 |
|  | Nov | 3 | 3 (100.00) |
|  | Dec | 10 | 1 (10.00) |
| 2018 | Jan | 10 | 0 |
|  | Feb | 6 | 0 |
|  | Mar | 6 | 1 (16.67) |
|  | Apr | 11 | 0 |
|  | May | 12 | 0 |
|  | June | 8 | 0 |
|  | July | 3 | 0 |
|  | Aug | 3 | 0 |
|  | Sep | 0 | 0 |
|  | Oct | 0 | 0 |
|  | Nov | 8 | 1 (12.50) |
|  | Dec | 23 | 2 (8.70) |
| 2019 | Jan | 14 | 0 |
|  | Feb | 30 | 1 (3.33) |
|  | Mar | 11 | 1 (9.90) |
|  | Apr | 9 | 1 (11.11) |
|  | May | 3 | 0 |
|  | June | 0 | 0 |
|  | July | 0 | 0 |
|  | Aug | 0 | 0 |
|  | Sep | 0 | 0 |
|  | Oct | 0 | 0 |
|  | Nov | 0 | 0 |
|  | Dec | 0 | 0 |
| 2020 | Jan | 0 | 0 |
|  | Feb | 0 | 0 |
|  | Mar | 0 | 0 |
|  | Apr | 0 | 0 |
|  | May | 0 | 0 |
|  | June | 0 | 0 |
|  | July | 0 | 0 |
|  | Aug | 13 | 0 |
|  | Sep | 5 | 0 |
|  | Oct | 8 | 0 |
|  | Nov | 6 | 0 |
|  | Dec | 21 | 1 (4.76) |
| 2021 | Jan | 41 | 9 (21.95) |
|  | Feb | 0 | 0 |
|  | Mar | 9 | 0 |
|  | Apr | 0 | 0 |
|  | May | 0 | 0 |
|  | June | 1 | 0 |
|  | July | 0 | 0 |
|  | Aug | 0 | 0 |
|  | Sep | 2 | 0 |
|  | Oct | 1 | 0 |
|  | Nov | 0 | 0 |
|  | Dec | 0 | 0 |
| 2022 | Jan | 0 | 0 |
|  | Feb | 8 | 0 |
|  | Mar | 21 | 0 |
|  | Apr | 18 | 8 (5.56) |
|  | May | 20 | 2 (5.00) |
|  | June | 0 | 0 |
|  | July | 7 | 2 (14.29) |
|  | Aug | 0 | 0 |
|  | Sep | 0 | 0 |
|  | Oct | 18 | 0 |
| Total |  | 531 | 50 (9.42) |
|  |  |  |  |
| 1. **Percentage of positive CBuVs by seasons** | | | |
| Seasons |  | Total samples | Number positive for CBuVs (%) |
| Winter (November-January) | | 183 | 19 (10.38) |
| Summer (February-May) | | 202 | 21 (10.39) |
| Rainy (June-October) | | 146 | 10 (6.84) |
| Total | | 531 | 50 (9.42) |

- Fisher exact test; p= 1 (p <0.05) not statistical significance when compared between Winter and Summer seasons
- Fisher exact test; p= 0.3288 (p <0.05) not statistical significance when compared between Winter and Rainy seasons
- Fisher exact test; p= 0.3405 (p <0.05) not statistical significance when compared between Summer and Rainy seasons

**Supplement Table 3.** Pairwise comparison of whole genome sequences of Thai CBuV (CU_FS231) with reference parvoviruses

| **Virus** | **Host** | **Accession number** | **Country** | **Year** | **Genome size** | **% Nucleotide identities (% Amino acid identities)** | | | |
| --- | --- | --- | --- | --- | --- | --- | --- | --- | --- |
|  |  |  |  |  |  | **WGS** | **NS1** | **VP1** | **VP2** |
| **This study** |  |  |  |  |  |  |  |  |  |
| CU_FS231 | Dog | OQ730242 | Thailand | 2017 | 4214 | 100.00  (100.00) | 100.00  (100.00) | 100.00  (100.00) | 100.00  (100.00) |
| CU_FS53 | Dog | OQ730240 | Thailand | 2016 | 4214 | 99.40  (99.90) | 99.10  (100.00) | 99.70  (99.90) | 99.80  (99.80) |
| CU_FS70 | Dog | OQ730241 | Thailand | 2016 | 1664 | N/A | N/A | N/A | 99.90  (100.00) |
| CU_FS232 | Dog | OQ730243 | Thailand | 2017 | 4214 | 100.00  (100.00) | 100.00  (100.00) | 100.00  (100.00) | 100.00  (100.00) |
| CU_FS235 | Dog | OQ730244 | Thailand | 2017 | 4214 | 100.00  (100.00) | 100.00  (100.00) | 100.00  (100.00) | 100.00  (100.00) |
| CU_FS236 | Dog | OQ730245 | Thailand | 2017 | 4214 | 100.00  (100.00) | 100.00  (100.00) | 100.00  (100.00) | 100.00  (100.00) |
| CU_FS20141 | Dog | OQ730246 | Thailand | 2017 | 4214 | 100.00  (100.00) | 99.90  (100.00) | 100.00  (100.00) | 100.00  (100.00) |
| CU_FS20932 | Dog | OQ730247 | Thailand | 2018 | 4214 | 99.80  (99.90) | 99.80  (99.80) | 99.70  (99.90) | 99.80  (99.80) |
| CU_FS22734 | Dog | OQ730248 | Thailand | 2018 | 4214 | 100.00  (100.00) | 100.00  (100.00) | 100.00  (100.00) | 100.00  (100.00) |
| CU_FS23266 | Dog | OQ730249 | Thailand | 2018 | 4214 | 99.50  (99.60) | 99.70  (99.80) | 99.30  (99.30) | 99.10  (99.30) |
| CU_FS23631 | Dog | OQ730250 | Thailand | 2019 | 839 | N/A | N/A | N/A | 100.00  (100.00) |
| CU_FS26352 | Dog | OQ730251 | Thailand | 2021 | 4214 | 99.80  (99.80) | 99.90  (100.00) | 99.60  (99.60) | 99.60  (99.70) |
| CU_FS26336 | Dog | OQ730252 | Thailand | 2021 | 2550 | N/A | N/A | 99.20  (99.40) | 99.50  (99.50) |
| CU_FS26340 | Dog | OQ730253 | Thailand | 2021 | 4214 | 99.60  (99.80) | 99.60  (99.70) | 99.60  (99.90) | 99.80  (99.80) |
| CU_FS26359 | Dog | OQ730254 | Thailand | 2021 | 2550 | N/A | N/A | 99.30  (99.40) | 99.60  (99.60) |
| CU_FS28678 | Dog | OQ730255 | Thailand | 2022 | 4214 | 95.20  (98.10) | 99.80  (100.00) | 91.00  (96.30) | 88.80  (95.40) |
| CU_FS28683 | Dog | OQ730256 | Thailand | 2022 | 4214 | 95.20  (98.00) | 99.80  (99.80) | 91.10  (96.30) | 88.90  (95.40) |
| CU_FS28696 | Dog | OQ730257 | Thailand | 2022 | 4214 | 99.70  (99.70) | 99.90  (100.00) | 99.40  (99.40) | 99.50  (99.50) |
| CU_FS28961 | Dog | OQ730258 | Thailand | 2022 | 4214 | 99.60  (99.70) | 99.70  (99.80) | 99.50  (99.60) | 99.50  (99.50) |
| CU_FS29327 | Dog | OQ730259 | Thailand | 2022 | 845 | N/A | N/A | N/A | 99.60  (99.60) |
| **Reference CBuV** |  |  |  |  |  |  |  |  |  |
| ITA/2015/297 | Dog | MF198244 | Italy | 2015 | 4219 | 99.70  (99.90) | 99.90  (100.00) | 99.60  (99.90) | 99.70  (99.80) |
| HUN/2012/22 | Dog | MF198245 | Hungary | 2012 | 4219 | 99.50  (99.70) | 99.70  (99.80) | 99.50  (99.70) | 99.60  (99.60) |
| HUN/2012/126 | Dog | MF198246 | Hungary | 2012 | 4219 | 99.60  (99.80) | 99.80  (100.00) | 99.50  (99.70) | 99.60  (99.60) |
| CBuV-88 | Dog | MH645362 | China | 2015 | 4249 | 99.40  (99.40) | 99.80  (100.00) | 98.90  (99.20) | 98.80  (98.90) |
| GXNN02-2018 | Dog | MK404087 | China | 2018 | 4219 | 99.20  (99.10) | 99.10  (98.10) | 99.50  (99.60) | 99.80  (100.00) |
| CaBuV/62/2017 | Dog | MT154050 | Italy | 2020 | 4219 | 99.20  (99.70) | 98.90  (99.40) | 99.60  (99.90) | 99.80  (100.00) |
| Henan38 | Dog | MT364251 | China | 2019 | 4219 | 99.60  (99.90) | 99.50  (99.80) | 99.80  (100.00) | 99.80  (100.00) |
| Henan44 | Dog | MT364252 | China | 2019 | 4219 | 99.50  (99.60) | 99.40  (99.40) | 99.60  (99.70) | 99.70  (99.80) |
| AH-001 | Dog | MT542982 | China | 2019 | 4219 | 99.20  (99.80) | 98.90  (99.50) | 99.70  (99.90) | 99.80  (100.00) |
| AH-002 | Dog | MT542983 | China | 2019 | 4219 | 99.50  (100.00) | 99.30  (100.00) | 99.70  (100.00) | 99.70  (100.00) |
| AH-003 | Dog | MT577645 | China | 2019 | 4219 | 98.80  (98.50) | 98.90  (99.50) | 98.80  (98.20) | 98.70  (97.90) |
| NWT-W25 | wolf | OK546094 | Canada | 2011 | 4234 | 99.00  (99.10) | 98.70  (99.10) | 99.40  (99.20) | 99.50  (99.10) |
| NWT-W116 | wolf | OK546096 | Canada | 2011 | 4234 | 99.00  (99.60) | 98.70  (99.20) | 99.60  (99.90) | 99.60  (99.80) |
|  |  |  |  |  |  |  |  |  |  |
| **Human and primate** |  |  |  |  |  |  |  |  |  |
| BF.86 | Human | JX027296 | Burkina Fas | 2009 | 4822 | 61.60  (55.10) | 62.60  (55.10) | 71.40  (64.40) | 70.50  (65.40) |
| Bufavirus 1-BF.7 | Human | JX027295 | Burkina Fas | 2008 | 4822 | 60.60  (54.50) | 62.50  (54.50) | 69.60  (64.00) | 69.20  (64.20) |
| Bufavirus 1-BJ133 | Human | KM580347 | China | 2014 | 4882 | 60.90  (55.20) | 62.90  (55.20) | 69.20  (63.60) | 68.90  (63.80) |
| Bufavirus 1-BF.96 | Human | JQ918261 | Burkina Fas | 2009 | 4912 | 60.60  (54.80) | 62.60  (54.80) | 69.60  (64.30) | 69.20  (64.50) |
| Bufavirus 2-BF.39 | Human | JX027297 | Burkina Fas | 2009 | 4562 | 60.50  (55.50) | 62.60  (55.50) | 67.50  (61.90) | 66.90  (61.40) |
| Bufavirus 3-AHP-74 | Human | AB982222 | Turkey | 2010 | 4745 | 60.70  (54.80) | 62.60  (54.80) | 67.70  (62.20) | 66.90  (61.90) |
| Bufavirus 3-BTN-310 | Human | AB847989 | Bhutan | 2011 | 4766 | 60.70  (55.10) | 62.50  (55.10) | 67.70  (62.50) | 66.90  (61.70) |
| Bufavirus 3-BTN-109 | Human | AB847988 | Bhutan | 2011 | 4734 | 60.80  (55.20) | 62.60  (55.20) | 67.70  (62.50) | 66.80  (61.70) |
| Wuharv parvovirus | Rhesus monkey | JX627576 | USA | 2010 | 4909 | 65.20  (60.50) | 67.10  (60.50) | 68.50  (68.60) | N/A |
| **Porcine and rat bufavirus** |  |  |  |  |  |  |  |  |  |
| GD015 | Swine | MK279317 | China | 2017 | 4189 | 45.60  (34.80) | 68.40  (66.80) | 71.80  (69.60) | 70.70  (69.50) |
| GD030 | Swine | MK279318 | China | 2017 | 4189 | 45.20  (34.90) | 67.80  (66.80) | 71.60  (68.90) | 70.60  (68.60) |
| GDHY-1 | swine | MK279319 | China | 2017 | 4189 | 45.50  (35.10) | 68.20  (67.40) | 71.20  (68.40) | 70.30  (68.60) |
| ZM38 | Crocidura hirta | NC026815 | Zambia | 2012 | 4613 | 43.90  (32.30) | 65.20  (61.50) | 57.80  (52.90) | 56.90  (53.30) |
| SY-2015 isolate 791102 | wild rat | NC028650 | China | 2014 | 4634 | 43.20  (31.80) | 66.00  (62.20) | 58.00  (53.90) | 56.30  (52.10) |
| **Feline bufavirus** |  |  |  |  |  |  |  |  |  |
| GD4 | Cat | MW970061 | China | 2020 | 594 | N/A | N/A | N/A | 100.00  (100.00) |
| GD6 | Cat | MW970062 | China | 2020 | 594 | N/A | N/A | N/A | 100.00  (100.00) |
| GD7 | Cat | MW970063 | China | 2020 | 594 | N/A | N/A | N/A | 100.00  (100.00) |
| GD8 | Cat | MW970064 | China | 2020 | 594 | N/A | N/A | N/A | 100.00  (100.00) |
| GD13 | Cat | MW970065 | China | 2020 | 594 | N/A | N/A | N/A | 100.00  (100.00) |
| GD18 | Cat | MW970066 | China | 2020 | 594 | N/A | N/A | N/A | 100.00  (100.00) |
| GD21 | Cat | MW970067 | China | 2020 | 594 | N/A | N/A | N/A | 100.00  (100.00) |
| GD64 | Cat | MW970069 | China | 2020 | 594 | N/A | N/A | N/A | 99.80  (100.00) |
| GD87 | Cat | MW970070 | China | 2020 | 594 | N/A | N/A | N/A | 99.80  (100.00) |
| GD 123 | Cat | MW970072 | China | 2020 | 594 | N/A | N/A | N/A | 99.80  (99.990) |
| GD 131 | Cat | MW970074 | China | 2020 | 594 | N/A | N/A | N/A | 99.80  (99.90) |
| **Bat Bufavirus** |  |  |  |  |  |  |  |  |  |
| Ms-PV/Shaanxi2011 | Bat | KC154061 | China | 2011 | 3403 | 61.30  (48.30) | 64.90  (61.10) | 64.90  (59.50) | 64.20  (58.70) |
| Mr-PV/Shaanxi201 | Bat | KC154060 | China | 2011 | 3891 | 61.50  (49.20) | 66.10  (61.40) | 65.70  (60.90) | 63.7  (58.50) |
| BtBV/V7/HUN/2013 | Bat | KR078344.1 | Hungary | 2013 | 3411 | 62.40  (49.00) | 67.2  (64.40) | 65.70  (60.80) | 65.60  (59.80) |
| **Other parvoviruses** |  |  |  |  |  |  |  |  |  |
| **Feline parvovirus** |  |  |  |  |  |  |  |  |  |
| FPV-kai.us.06 | Cat | EU659115.1 | USA | 2006 | 4269 | 57.80  (43.80) | 57.00  (45.80) | 52.70  (37.90) | 51.70  (35.00) |
| FPV-4.us_64 | Cat | EU659112.1 | USA | 1964 | 4269 | 57.80  (43.70) | 57.10  (45.60) | 52.60  (38.10) | 51.60  (35.00) |
| **Canine parvovirus** |  |  |  |  |  |  |  |  |  |
| CPV-2C -CU 24 | Dog | MH711894. | Thailand | 2016 | 4269 | 58.00  (43.80) | 57.10  (45.80) | 52.70  (37.80) | 51.50  (34.90) |
| CPV-5.us.79 | Dog | EU659116 | USA | 1979 | 4269 | 57.90  (43.70) | 57.10  (45.80) | 52.60  (37.90) | 51.40  (34.90) |
| **Canine bocavirus** |  |  |  |  |  |  |  |  |  |
| CBoV-2-GZHD15 | Dog | KY038922. | China | 2015 | 5041 | 37.80  (20.20) | 39.50  (21.70) | 42.30  (21.10) | 40.60  (16.50) |
| CBoV-2-17CC0312 | Dog | MH626633 | China | 2017 | 5069 | 38.50  (20.80) | 39.20  (21.70) | 42.90  (21.50) | 41.20  (16.70) |
| CBoV-1-HK831F | Dog | JQ692591 | China | 2010 | 5054 | 39.00  (21.30) | 40.70  (21.70) | 43.30  (21.50) | 41.40  (16.70) |

**Supplement Table 4.** Genetic analysis of VP2 gene between Thai CBuVs and reference CBuVs.

| **Virus** | **Country/Year** | **Accession number** | **VP2 gene** | | | | | | | | | | | | | | | |
| --- | --- | --- | --- | --- | --- | --- | --- | --- | --- | --- | --- | --- | --- | --- | --- | --- | --- | --- |
|  |  |  | **54** | **70** | **91** | **93** | **175** | **228** | **230** | **236** | **264** | **271** | **302** | **370** | **418** | **426** | **477** | **508** |
| **Type A** |  |  |  |  |  |  |  |  |  |  |  |  |  |  |  |  |  |  |
| CU_FS53 | Thailand/2016 | OQ730240 | T | S | Q | Q | L | G | Q | K | D | I | E | T | S | A | K | A |
| CU_FS231 | Thailand/2017 | OQ730242 | . | . | . | . | . | . | . | . | . | . | . | . | . | . | . | . |
| CU_FS232 | Thailand/2017 | OQ730243 | . | . | . | . | . | . | . | . | . | . | . | . | . | . | . | . |
| CU_FS235 | Thailand/2017 | OQ730244 | . | . | . | . | . | . | . | . | . | . | . | . | . | . | . | . |
| CU_FS236 | Thailand/2017 | OQ730245 | . | . | . | . | . | . | . | . | . | . | . | . | . | . | . | . |
| CU_FS20141 | Thailand/2017 | OQ730246 | . | . | . | . | . | . | . | . | . | . | . | . | . | . | . | . |
| CU_FS20932 | Thailand/2018 | OQ730247 | . | . | . | . | . | . | . | . | . | . | . | . | . | . | . | . |
| CU_FS22734 | Thailand/2018 | OQ730248 | . | . | . | . | . | . | . | . | . | . | . | . | . | . | . | . |
| CU_FS23266 | Thailand/2018 | OQ730249 | . | . | . | . | . | E | . | . | . | . | . | . | . | . | . | . |
| CU_FS26352 | Thailand/2021 | OQ730251 | . | . | . | . | . | . | . | . | . | . | . | . | . | . | . | . |
| CU_FS26340 | Thailand/2021 | OQ730253 | . | . | . | . | . | . | . | . | . | . | . | . | . | . | . | . |
| CU_ FS28696 | Thailand/2022 | OQ730257 | . | . | . | . | . | . | . | . | . | . | . | . | . | . | . | . |
| CU_ FS28961 | Thailand/2022 | OQ730258 | . | . | . | . | . | . | . | . | . | . | . | . | . | . | . | . |
| Henan38 | China/2019 | MT364251 | . | . | . | . | . | . | . | . | . | . | . | . | . | . | . | . |
| Henan44 | China/2019 | MT364252 | . | . | . | . | . | . | . | . | . | . | . | . | . | . | . | . |
| GXNN01 | China/2018 | MK404086 | . | . | . | . | . | . | . | . | . | . | . | . | . | . | . | . |
| GXNN02 | China/2018 | MK404087 | . | . | . | . | . | . | . | . | . | . | . | . | . | . | . | . |
| CBuV-88 | China/2015 | MH645362 | . | . | . | . | . | . | . | . | . | . | . | . | . | . | . | . |
| AH-001 | China/2019 | MT542982 | . | . | . | . | . | . | . | . | . | . | . | . | . | . | . | . |
| AH-002 | China/2019 | MT542983 | . | . | . | . | . | . | . | . | . | . | . | . | . | . | . | . |
| AH-003 | China/2019 | MT577645 | . | . | . | . | . | . | . | . | . | . | . | . | . | . | . | . |
| Dog/62 | Italy/2017 | MT154050 | . | . | . | . | . | . | . | . | . | . | . | . | . | . | . | . |
| Dog/297 | Italy/2015 | MF198244 | . | . | . | . | . | . | . | . | . | . | . | . | . | . | . | . |
| Dog/22 | Hungary/2012 | MF198245 | . | . | . | . | . | . | . | . | . | . | . | . | . | . | . | . |
| Dog/126 | Hungary/2012 | MF198246 | . | . | . | . | . | . | . | . | . | . | . | . | . | . | . | . |
| NWT-W25 | Canada/2011 | OK546094 | . | . | . | . | . | . | . | . | N | . | . | . | . | . | . | . |
| NWT-W116 | Canada/2011 | OK546096 | . | . | . | . | . | . | . | . | . | . | . | . | . | . | . | . |
| **Type B** |  |  |  |  |  |  |  |  |  |  |  |  |  |  |  |  |  |  |
| FS28678 | Thailand/2022 | OQ730255 | I | T | T | H | I | Q | E | Q | I | N | V | S | T | D | N | G |
| FS28683 | Thailand/2022 | OQ730256 | I | T | T | H | I | Q | E | Q | I | N | V | S | T | D | N | G |
| 9AS | Italy/20015 | MT154051 | I | T | T | H | I | Q | E | Q | I | N | V | S | T | D | N | G |
| 35 | Italy/2016 | MT154052 | I | T | T | H | I | Q | E | Q | I | N | V | S | T | D | N | G |

**Supplement Table 5.** Prediction of antigenic epitopes of Thai CBuV strain CU_FS20141 and CU_FS28678

| 1. Prediction of antigenic epitopes of Thai CBuV strain CU_FS20141 | | | | |
| --- | --- | --- | --- | --- |
| **Rank** | **Location** | **Epitope** | **Score** | **Recommend*** |
| 1 | 239 - 258 | KFDDIQFITVENCVPIELLR | 1.000 | 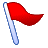 |
| 2 | 99 - 118 | NDSYHAKVETPWSLLHANCW | 0.845 | 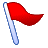 |
| 3 | 42 - 61 | NRTEFHYHNGEVTIVCHATR | 0.775 |  |
| 4 | 301 - 320 | GEGQNFEMVNTWQWGDRDTP | 0.762 |  |
| 5 | 373 - 392 | ASEKAVFDYAHGEMSPNEKD | 0.724 |  |
| 6 | 504 - 523 | IVTYATFWWSGTLVFKGKLR | 0.582 |  |
| 7 | 322 - 341 | AAATKVDNFHIGYQWPEWHF | 0.532 |  |
|  |  |  |  |  |
| 1. Prediction of antigenic epitopes of Thai CBuV strain CU_FS28678 | | | | |
| **Rank** | **Location** | **Epitope** | **Score** | **Recommend*** |
| 1 | 90 - 109 | QTLHGRDTINDSYHAKVETP | 1.000 | 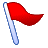 |
| 2 | 239 – 258 | KYDDIQFITVENCVPIELLR | 0.855 | 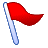 |
| 3 | 301 - 320 | GVGQNFEMVNTWQWGDRDTP | 0.587 |  |
| 4 | 25 - 44 | GGGGGGSGVGHSTGAFNNRT | 0.531 |  |
| 5 | 322 - 341 | AAATRVNNFHIGYQWPEWHF | 0.454 |  |
| 6 | 370 - 389 | STGASEKAVFDYNHGEMSPN | 0.444 |  |
| 7 | 116 - 135 | NCWGVWFNPADFQHMTAICN | 0.356 |  |

**Supplement Table 6.** Evidence for positive and negative selection using various detection methods for CBuVs

| **BuV**  **genome** | **Selection pressure analysis** | **Program** | | |
| --- | --- | --- | --- | --- |
|  |  | **FEL** | **FUBAR** | **MEME** |
| NS1 | Positive selection | 0 | 3  (112, 116, 512) | 7  (207, 382, 415, 512, 514, 515, 600) |
|  | Negative | 160 | 184 |  |
|  | Threshold | 0.1 | posterior probability of 0.9 | 0.1 |
|  | Rate distribution |  |  | 0.0827 |
| VP1 | Positive selection | 1  (256) | 1  (256) | 8  (28, 78, 149, 199, 256, 430, 500, 660) |
|  | Negative | 161 | 198 |  |
|  | Threshold | 0.1 | posterior probability of 0.9 | 0.1 |
|  | Rate distribution |  |  | 0.0971 |
| VP2 | Positive selection | 2 (22, 113) | 2 (22,113) | 5 (14, 56, 248, 517, 557) |
|  | Negative | 203 | 221 |  |
|  | Threshold | 0.1 | posterior probability of 0.9 |  |
|  | Rate distribution |  |  | 0.103 |

**Supplement Table 7.** Recombinant analysis of Thai CBuV strain CU_FS28678

Major parent: Thai CBuV strain CU_FS28696 (CBuV-subgroup A)

Minor parent: CBuV strain 9AS from Italy (MT154051; CBuV-subgroup B)

| **Methods** | **Av P-val** |
| --- | --- |
| RDP | 1.067 x 10 ^-07^ |
| GENECONV | 5.806 x 10 ^-06^ |
| BootScan | 4.323 x 10 ^-05^ |
| Maxchi | 1.144 x 10 ^-09^ |
| Chimaera | 8.612 x 10 ^-03^ |
| SiScan | 7.014x 10 ^-30^ |
| 3Seq | 2.222x 10 ^-21^ |

**Supplement table 8.** Nucleotide sequences of primers used for CBuV detection and sequencing in this study.

| **Primer name** | **Nucleotide sequence (5’-3’)** | **Position *** | **Product size** | **Reference** |
| --- | --- | --- | --- | --- |
| **Primer detection** |  |  |  |  |
| CPPV 165F | CTGGTTTAATCCAGCAGACT | 2923-2942 | 208 | ^1^ |
| CPPV 371R | TGAAGACCAAGGTAGTAGGT | 3111-3130 |  |  |
| **Primer sequencing** |  |  |  |  |
| CBV_NV1F | GCAGTAAATTTGATACTTCATCTTG | 1-25 | 1052 | This study |
| BUFA-R1 | GCTTTGATGATAGTTTCACCACCTG | 1028-1052 |  | ^1^ |
| CBV_N2F | ACACAAGAAAAATGGATGCTAGGAG | 970-994 | 720 | This study |
| CBV_N2R | CTATTTTTGAGTTCAATTCTGACACA | 1601-1626 |  | This study |
| CBV_N3F | CAGACTTGATCAAAAAGGCAAAGGCAGC | 1461-1488 | 731 | This study |
| CBV_N3R | GTAATTGTAGCCAGGAGGAACCCAACC | 2165-2191 |  | This study |
| CBV_N4F | GCCTCCGACTAATCGACCACG | 1957-1977 | 1200 | This study |
| CBV_N4R | CCTAGAGAGTCTATGTACATRTTGTC | 3131-3156 |  | This study |
| CBV_N5F | TGGGGAGTCTGGTTTAATCCAGC | 2915-2937 | 688 | This study |
| CBV_N6R | CACCACTGCTGTAGTGGAAGTGCCA | 3578-3602 |  | This study |
| CBV_N7F | CTCAAYTAGAATACCACTGGCAATCT | 3387-3412 | 496 | This study |
| CBV_N7R | GTGAATGGTCCGAATGTGTTGTGGTATG | 3855-3882 |  | This study |
| CBV_N8F | CAACAAATGACAGGACAAACAGACTACAC | 3759-3786 | 512 | This study |
| BUFA-R5 | TTATAGAGTAATATTAGGCATAGCT | 4246-4270 |  | ^1,2^ |

*Position based on CBuV strain CBuV-88/China/2015_(MH645362)

**Supplement Figure**

Supplement Figure 1 Location of sample collection and CBuVs detected by provinces in this study (Map of Thailand with permission by World Trade Press)

Supplement Figure 2 Genome structure of Canine parvoviruses and BuVs

Supplement Figure 3 Phylogenetic analysis of VP1 gene of Thai CBuV, the phylogenetic tree was constructed by using MEGA v.7.0 with neighbor-joining algorithm with Kimura-2 parameter model with 1,000 replications of bootstrap analysis. The pink circle represents Thai CBuVs.

Supplement Figure 4 Phylogenetic analysis of VP2 gene of Thai CBuV, the maximum likelihood tree constructed by using IQ-TREE version 2.1.3 (http://www.iqtree.org/) using the TVMe +IG4 model of nucleotide substitution, default heuristic search options, and ultrafast bootstrapping with 1000 replicates. Tree was visualized by iTOL version 6.0 (<https://itol.embl.de/>). The pink circles indicate the Thai CBuVs.


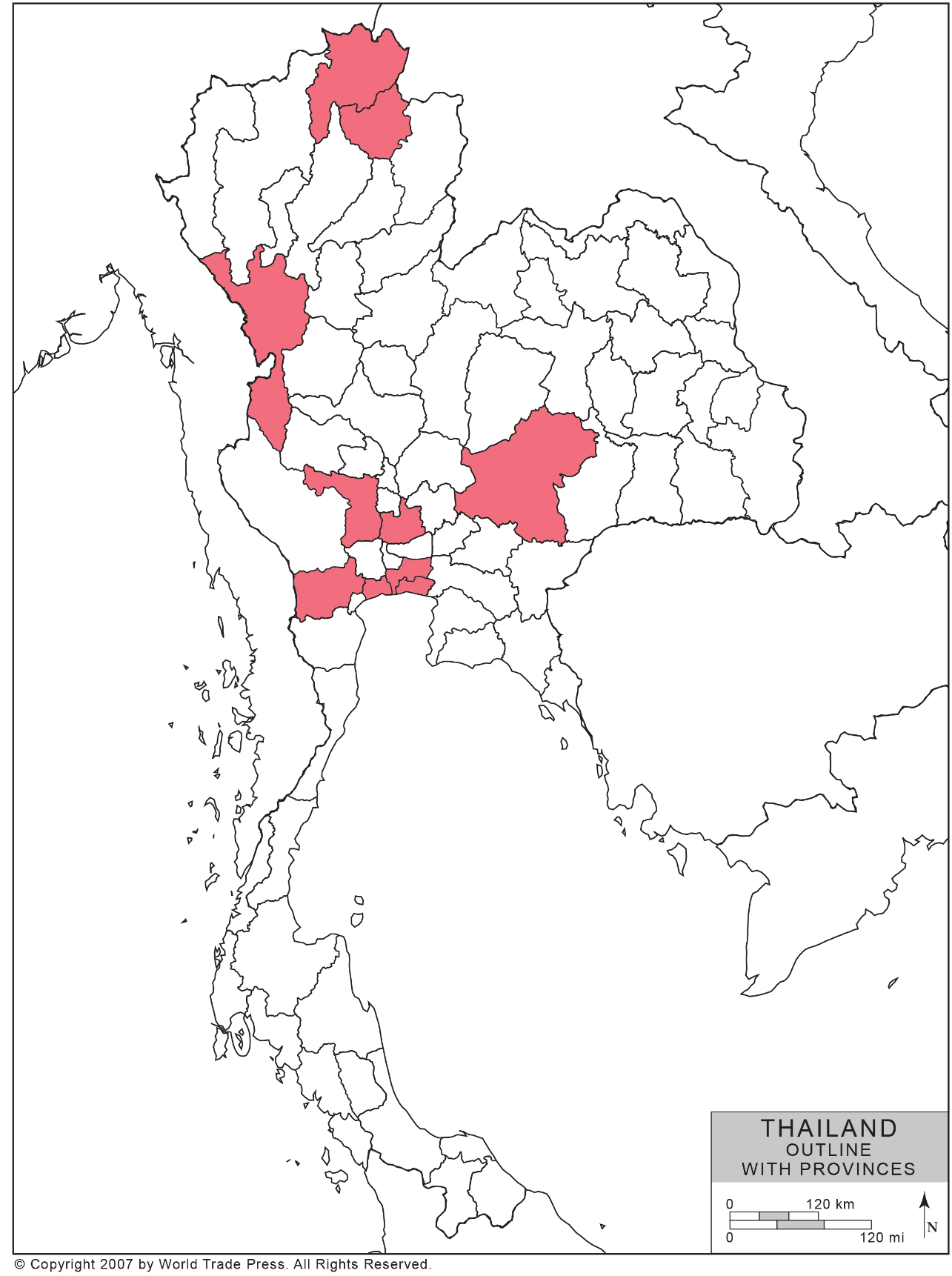
 **Supplement Figure 1** Location of sample collection and CBuVs detected by provinces in this study (Map of Thailand with permission by World Trade Press)


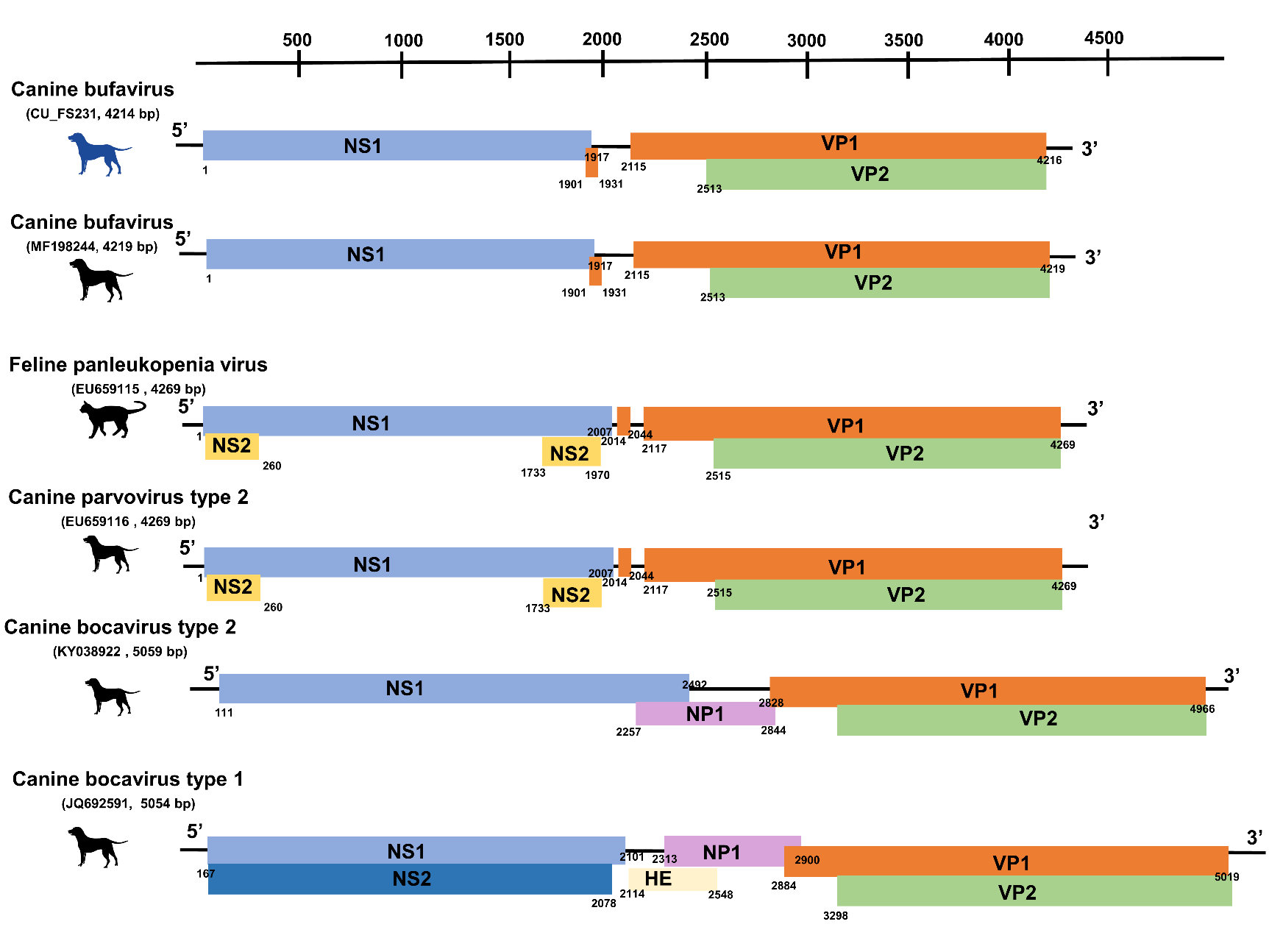
 **Supplement Figure 2** Genome structure of Canine parvoviruses & BuV

**
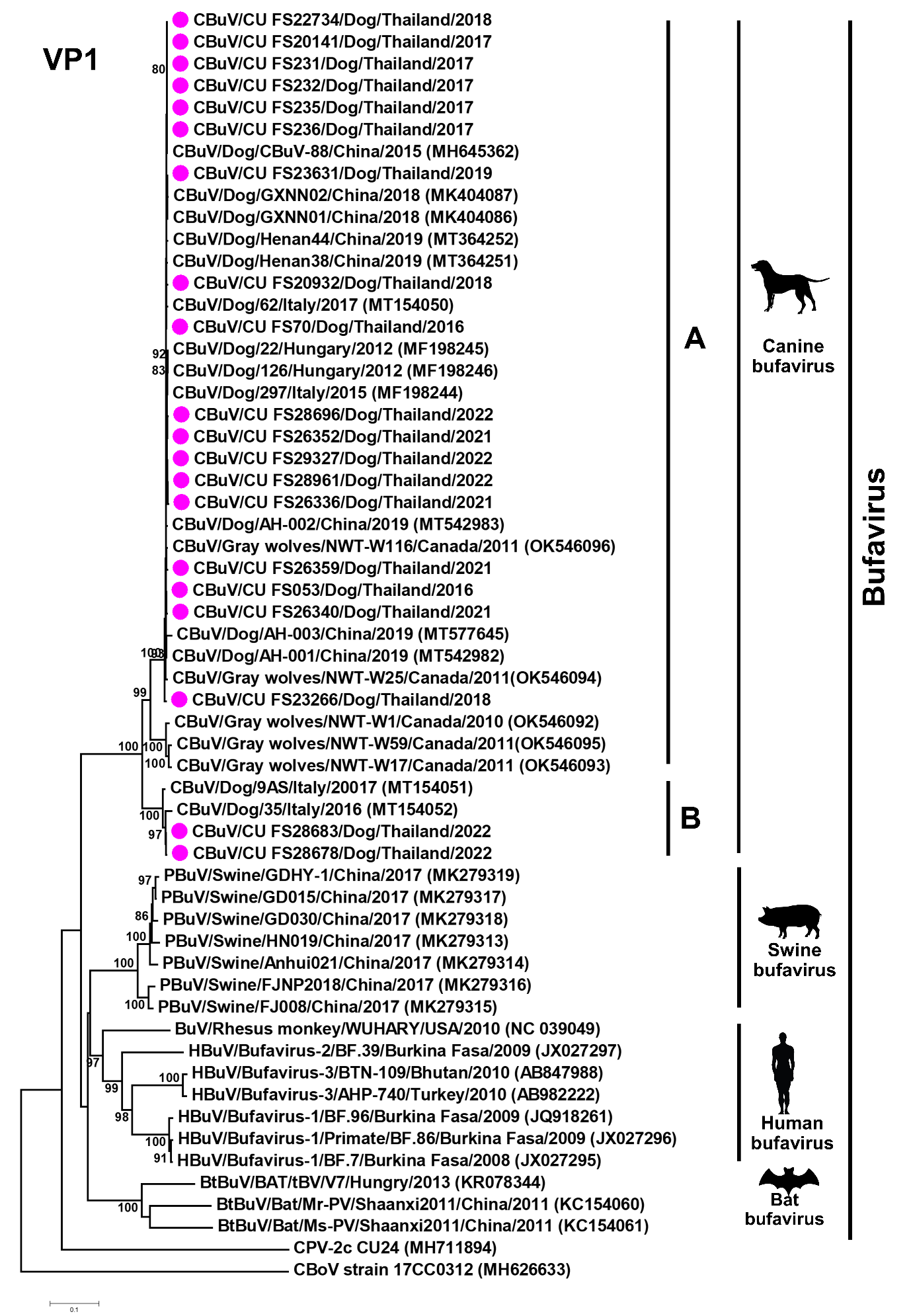
Supplement Figure 3.** Phylogenetic analysis of VP1 gene of Thai CBuV

**Supplement Figure 4.** Phylogenetic analysis of VP2 gene of Thai CBuV


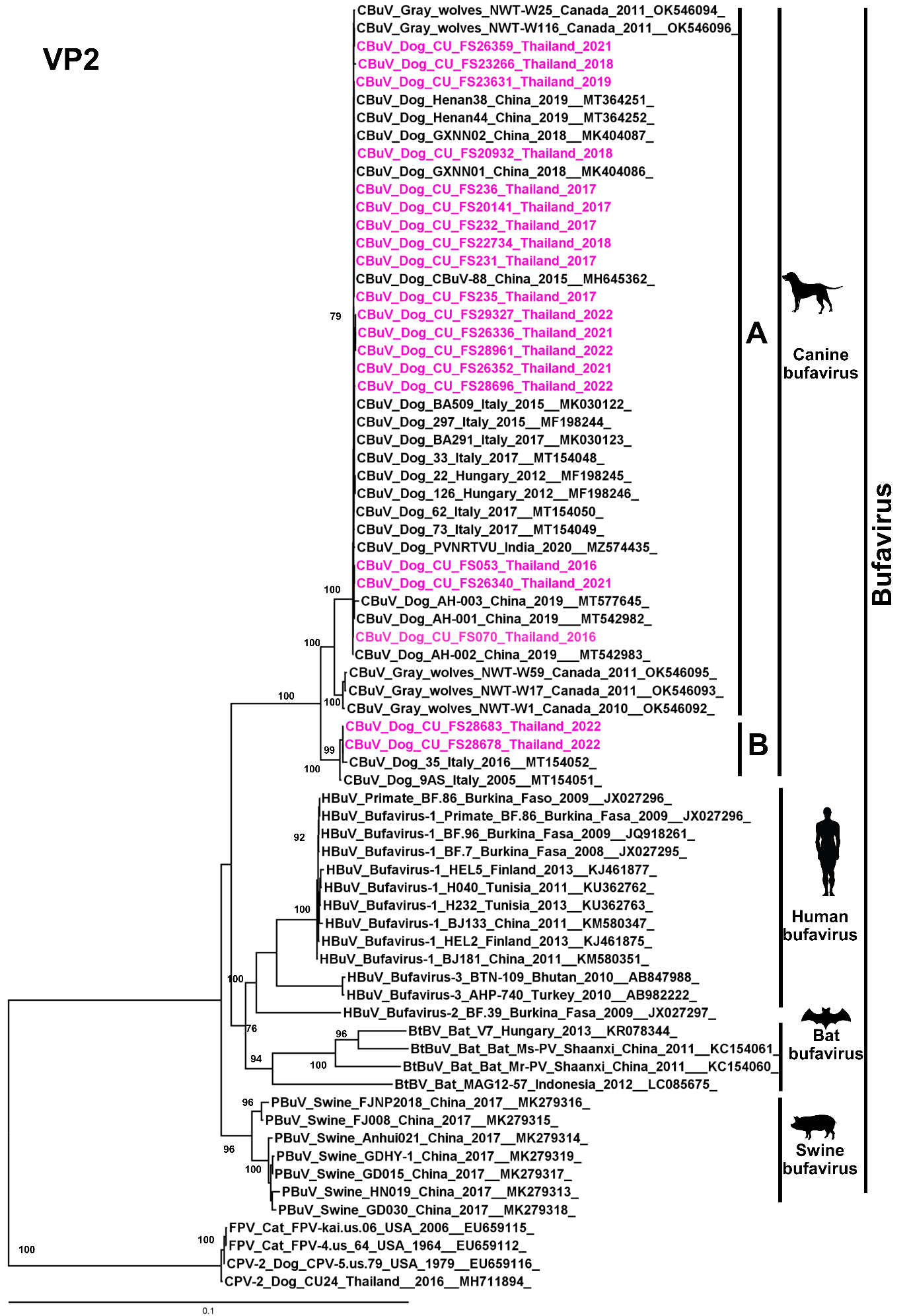


**Reference**

1 Wang, Y. *et al.* Genetic and phylogenetic analysis of canine bufavirus from Anhui Province, Eastern China. *Infection, Genetics and Evolution* **86**, 104600 (2020).

2 Sun, W. *et al.* First identification of a novel parvovirus distantly related to human bufavirus from diarrheal dogs in China. *Virus Res* **265**, 127-131, doi:10.1016/j.virusres.2019.03.020 (2019).
